# The European Forest Condition Monitor: Using Remotely Sensed Forest Greenness to Identify Hot Spots of Forest Decline

**DOI:** 10.3389/fpls.2021.689220

**Published:** 2021-12-01

**Authors:** Allan Buras, Anja Rammig, Christian S. Zang

**Affiliations:** ^1^Land Surface-Atmosphere Interactions, Technische Universität München, Freising, Germany; ^2^Forests and Climate Change, Hochschule Weihenstephan-Triesdorf, Freising, Germany

**Keywords:** MODIS NDVI, drought, late frost, phenology, water stress, ice storm, windthrow

## Abstract

Forest decline, in course of climate change, has become a frequently observed phenomenon. Much of the observed decline has been associated with an increasing frequency of climate change induced hotter droughts while decline induced by flooding, late-frost, and storms also play an important role. As a consequence, tree mortality rates have increased across the globe. Despite numerous studies that have assessed forest decline and predisposing factors for tree mortality, we still lack an in-depth understanding of (I) underlying eco-physiological mechanisms, (II) the influence of varying environmental conditions related to soil, competition, and micro-climate, and (III) species-specific strategies to cope with prolonged environmental stress. To deepen our knowledge within this context, studying tree performance within larger networks seems a promising research avenue. Ideally such networks are already established during the actual period of environmental stress. One approach for identifying stressed forests suitable for such monitoring networks is to assess measures related to tree vitality in near real-time across large regions by means of satellite-borne remote sensing. Within this context, we introduce the European Forest Condition monitor (EFCM)—a remote-sensing based, freely available, interactive web information tool. The EFCM depicts forest greenness (as approximated using NDVI from MODIS at a spatial resolution of roughly 5.3 hectares) for the pixel-specific growing season across Europe and consequently allows for guiding research within the context of concurrent forest performance. To allow for inter-temporal comparability and account for pixel-specific features, all observations are set in relation to normalized difference vegetation index (NDVI) records over the monitoring period beginning in 2001. The EFCM provides both a quantile-based and a proportion-based product, thereby allowing for both relative and absolute comparison of forest greenness over the observational record. Based on six specific examples related to spring phenology, drought, late-frost, tree die-back on water-logged soils, an ice storm, and windthrow we exemplify how the EFCM may help identifying hotspots of extraordinary forest greenness. We discuss advantages and limitations when monitoring forest condition at large scales on the basis of moderate resolution remote sensing products to guide users toward an appropriate interpretation.

## Introduction

The higher frequency of more extreme climate conditions in course of climate change poses certain threats on forests worldwide (Allen et al., 2010, 2015; Anderegg et al., 2015; Vitasse et al., 2019; Buras et al., 2020; Kannenberg et al., 2020). Among potential direct causes of the resulting forest decline and tree mortality are in particular so-called hotter droughts (Allen et al., 2010, 2015; Buras et al., 2018; Brun et al., 2020; Rita et al., 2020; Schuldt et al., 2020), late-frost (Menzel et al., 2015; Príncipe et al., 2017; Vitasse et al., 2019), periodically water-logged soils after floods and heavy precipitation (Kreuzwieser and Rennenberg, 2014), ice storms (Roženbergar et al., 2020), and winter storms (Valinger et al., 2014). Since extreme environmental conditions often impair tree defense mechanisms, secondary pathogens such as fungi and beetles frequently amplify tree decline and die-back rates (Bigler et al., 2006; Kohler et al., 2010; Spruce et al., 2019).

Although the underlying environmental causes of observed forest decline often can be identified, the individualistic response of trees to extreme environmental conditions remains largely unexplained. Therefore, we currently lack a mechanistic explanation of frequently observed individual growth responses to adverse climatic conditions which may ultimately result in neighboring dying and surviving trees of the same species (Cailleret et al., 2017). Even though first assessments have identified soil properties, stand structure, and micro-climate as influencing factors of forest decline and tree mortality (Lévesque et al., 2013; Rehschuh et al., 2017; Buras et al., 2018), the eco-physiological processes that govern the fate of single trees still need further investigation (Bascietto et al., 2018; D'Andrea et al., 2019; Dox et al., 2020, 2021; Schuldt et al., 2020). One reason for this important research gap is related to the fact that key eco-physiological processes such as sap flow, regulation of leaf water potential, and changes in xylem conductivity cannot be studied retrospectively for dead trees. At the same time, it is difficult to forecast where, when, and which trees are going to die in order to install monitoring equipment prior to death.

An important approach to deepen our knowledge on forest decline is the evaluation of large-scale tree-ring networks which allows for a retrospective analysis of secondary tree growth and its relation to environmental factors. For instance, the effects of droughts on tree growth have been studied using large tree-ring networks, which suggested so-called drought legacies, i.e., prolonged reductions of secondary growth after drought, to render a frequently observed phenomenon (Anderegg et al., 2015; Kannenberg et al., 2018, 2020; Gazol et al., 2020). More specific tree-ring networks targeting both dead and live trees revealed that dying trees often featured decreased growth rates if compared to surviving trees up to several decades prior to death (Camarero et al., 2015; Pellizzari et al., 2016; Cailleret et al., 2017; Buras et al., 2018). Another important source of information within this context comes from long-term ecological monitoring and the evaluation of eddy covariance data from flux towers within large networks [e.g., long-term ecological research (LTER), integrated carbon observation system (ICOS), and FLUXNET] which allow for quantifying declining net biome production under extreme climatic conditions (e.g., Ciais et al., 2005; Bastos et al., 2020; Pastorello et al., 2020).

Despite the value of the aforementioned retrospective investigations, a deeper process-based understanding of forest decline can only be achieved by intensively monitoring stressed and/or dying trees throughout the stress period and the process of death (Bascietto et al., 2018; D'Andrea et al., 2019; Dox et al., 2020, 2021; Scharnweber et al., 2020; Schuldt et al., 2020). As mentioned above, this however requires timely installation of monitoring equipment. Consequently, the majority of our knowledge about tree eco-physiology under extreme conditions is based on experimental treatments of saplings (e.g., Seidel and Menzel, 2016; Seidel et al., 2016; Ruehr et al., 2019; Seo et al., 2020).

To increase the available data for studying forest decline, it is necessary to identify hotspots of forest stress in space and time, ideally, in near real-time to allow for contemporary investigations under stressful conditions. Satellite-borne remote sensing renders a means to obtain such information on a large scale. That is, satellites provide information on vegetation surface reflectance which can be translated into vegetation indices and/or products that serve as proxies for tree condition and productivity (Kogan, 1995; Anyamba and Tucker, 2012; Misra et al., 2016; Rogers et al., 2018; Liu et al., 2019; Spruce et al., 2019; Brun et al., 2020; Buras et al., 2020; Rita et al., 2020; Shekhar et al., 2020). If corresponding satellite imagery is regularly updated and obtained at fairly high spatiotemporal resolution (i.e., high enough to resolve single forest stands at weekly intervals) over decades to provide sufficiently long referencing periods, such data can be used to generate products that allow for a near-real time monitoring of forest condition (but see section Limitations for limitations of this approach).

Within this context, we here introduce a framework for monitoring European forest condition, the European Forest Condition Monitor (EFCM), a freely available web information tool which is based on Terra Moderate Resolution Imaging Spectroradiometer Normalized Difference Vegetation Index (MODIS NDVI, see section Computation of Central Metrics). The EFCM provides relative measures of forest greenness at a spatial resolution of 231.25 × 231.25 m at 16-day intervals. The main purpose of the EFCM is to provide forest researchers, stakeholders, and policy makers with large scale information on forest condition allowing for identifying hotspots of extraordinary canopy greenness. Based on the information provided by the EFCM, additional ground-based investigations and monitoring campaigns may help to elucidate the causes underlying extraordinary canopy greenness, thereby potentially deepening our knowledge on the eco-physiology of trees under stress within a natural setting. In the following, we describe the methodological approach of the EFCM and exemplify its application using specific case studies related to phenology, drought, late-frost, flooding, ice storms, and windthrow.

## Materials and Methods

### Computation of Central Metrics

The EFCM is based on 16-day maximum value composites derived from daily Terra MODIS NDVI observations spanning the period of 2001 to current at a spatial resolution of 231.25 × 231.25 m and covering the area of Europe. We based the EFCM on NDVI since it represents vegetation greenness which reflects tree-species phenology (Misra et al., 2016, 2018) and responds sensitively to drought-stress in trees (Anyamba and Tucker, 2012; Orth et al., 2016; Buras et al., 2018, 2020; Brun et al., 2020; Rita et al., 2020), late-frost damage (Rubio-Cuadrado et al., 2021), tree die-back (Rogers et al., 2018; Liu et al., 2019; Spruce et al., 2019), and has been used to compute vegetation condition indices (e.g., Kogan, 1995). Given its wide application within a forest-decline context, we consider NDVI as a meaningful proxy for actual forest condition which, in case of extreme values, may represent extraordinary phenology (early start of the season and early senescence, e.g., Misra et al., 2016; Brun et al., 2020) or canopy greenness due to atypical environmental conditions such as drought, late-frost, water-logged soils succeeding long-lasting precipitation events, ice storms, and windthrow (e.g., Bascietto et al., 2018; Buras et al., 2020; Rita et al., 2020). NDVI is computed according to Equation 1 (see also Rouse et al., 1974):


(1) $$\begin{array}{r} NDVI=\frac{NIR-RED}{NIR+RED} \end{array}$$

with NIR representing the surface reflectance in the near infrared spectrum and RED representing the surface reflectance in the red spectrum. Given its computation, NDVI ranges from −1 to 1 with (1) negative values representing clouds, water, and snow, (2) values around zero representing bare rock and soil, while (3) values above 0.5 typically represent densely vegetated surfaces such as forests or crops (Lillesand et al., 2014). NDVI and pixel quality information layers are obtained from the Application for Extracting and Exploring Analysis Ready Samples (AppEEARS)^1^. The preparation of NDVI time series is based on previous studies (Misra et al., 2016, 2018; Buras et al., 2020) and will be described in the following.

For masking MODIS NDVI of non-forest areas, we use the Coordinated Information on the European Environment land cover map (CORINE, Büttner et al., 2004) retrieved from COPERNICUS^2^ at a spatial resolution of 100 m and remapped to the MODIS projection using the nearest neighbor method to retain the original classes. The retrieved MODIS layers are masked to only represent pixels which according to the different time-steps of CORINE land-cover since the year 2000 (i.e., 2000, 2006, 2012, and 2018) consistently refer to the land-cover classes of broadleaved forest, coniferous forest, and mixed forest, thereby lowering the probability of artifacts from land-use change.

The MODIS pixel quality layers are used to mask NDVI-pixels of the corresponding time-step that represent poor quality to keep the influence of snow and clouds on NDVI time series as low as possible. Thus, only pixels with good and marginal quality are retained for further analyses. Due to this masking, pixel-specific time series usually contain gaps which are linearly interpolated from the neighboring values. Since the gap-filled time series may still contain pixels with insufficient quality that is usually reflected in extraordinarily low NDVI values, we subsequently identify and remove negative outliers. Outliers are defined as those exceeding two negative standard deviations of standardized differences between interpolated NDVI time series and a corresponding Gaussian-filtered time series whose filter size was chosen to be 80 days, i.e., 5 time steps (Misra et al., 2016). Removed outliers are replaced by the corresponding values of the Gaussian-filtered time series. Eventually, the quality-checked, gap-filled, and outlier-removed NDVI time series is smoothed by applying the same Gaussian filter with a window size of five time steps. The resulting time series are finally detrended individually, i.e., pixel-specific to remove long-term trends that have been reported for vegetation indices (Bastos et al., 2017; Buras et al., 2020).

To represent relative forest condition for specific days of the year (DOYs) over the whole observational period (2001-current), we, for each pixel and time step, computed the corresponding quantile *Q*. That is, each pixel-specific time-series was transformed into quantiles by assigning each value its corresponding rank within all observations of this pixel and dividing it by the number of observations:


(2) $$\begin{array}{r} Q_{t}=\frac{R\left(NDVI_{t}\right)}{n}-\frac{1}{n\cdot 2} \end{array}$$

with *R* representing the relative rank of the NDVI at a given time step *t* and *n* being the number of total observations. The subtraction by 1/(*n* × 2) is applied to center the quantiles around 0.5. Consequently, *Q*_*t*_ values range from 1/(*n* × 2) to 1–1/(*n* × 2). While a value of 1/(*n* × 2) represents the absolutely lowest observation over the period of investigation (currently 2001–2020) for a given DOY at a given pixel, the value 1-1/(*n* × 2) indicates the absolutely highest observation. This metric is closely related to the vegetation condition index (VCI, Kogan, 1995). However, it does not have the disadvantage of the VCI to become heavily skewed in case extreme values enter the distribution. That is, since VCI relies on the range of NDVI over the observational period, extraordinarily low or high NDVI values can lead to heavily skewed distributions, whereas the computation of quantiles results in uniformly distributed observations.

In addition, since a rank-based measure as *Q* does not allow for comparing absolute changes in NDVI among pixels, we computed proportional deviations from the median (*PDM*) for each pixel-DOY combination according to Equation 3:


(3) $$\begin{array}{r} PDM_{t}=\frac{NDVI_{t}-\bar{NDVI}}{\bar{NDVI}}-1 \end{array}$$

With the bar over NDVI indicating the median over all observations. Consequently, *PDM* represents a proportional deviation from the median and thus allows for better comparison among pixels since it is not related to the range of local NDVI observations as is the case with *Q*. Thus, *PDM* can be considered a metric which complements *Q* since it allows for depicting absolute variations in comparison to ranks as is the case with *Q*. We applied these two standardization methods to account for pixel-specific stand characteristics (e.g., tree-species, soil type) that result in pixel-specific statistical properties of NDVI time-series. While doing so, we opted for non-parametric estimates of forest condition since parametric estimates, for instance, a z-transformation, rely on parameter estimates (in case of z-transformation mean and standard deviation) which assume an underlying normal distribution which is not fulfilled for NDVI that features a bounded distribution (Buras et al., 2020). Due to this standardization procedure, differences among pixels regarding their absolute NDVI are masked. Consequently, spatial variations in *Q* and *PDM* should be interpreted as pixel-specific, relative differences in forest greenness over time and not as absolute variations in forest greenness among pixels.

We constrained the visualization of *Q* and *PDM* to the pixel-specific growing season to account for differing lengths of growing seasons across Europe. In order to define the pixel-specific growing season, we used high resolution (1 km^2^) CHELSA (Climatologies at high resolution for the earth's land surface areas) monthly mean temperature climatologies (Karger et al., 2017) which were remapped to MODIS resolution and linearly interpolated to daily climatologies for each pixel. For each pixel the period with a temporally interpolated mean temperature above 5°C was defined as the growing season. The threshold of 5°C is based on identified thresholds for cambial activity, xylogenesis, and growth (Rossi et al., 2007; Körner, 2015).

For the visualization, we use color charts ranging from red, over orange, yellow, dark-gray, light-blue, and dark blue representing a gradient from low to high values of *Q* and *PDM*, respectively. Thus, while reddish colors represent relatively low forest greenness, blueish colors refer to high greenness. Light-gray colors indicate pixels outside the pixel-specific growing season. For *Q*, the color chart is resolved in steps representing the number of expected quantiles. That is, for the current observation period spanning 20 years (2001–2020), the color-chart is resolved into 21 classes according to 20 breaks ranging from 1/40 to 1–1/40 in steps of 1/20. For *PDM*, we subjectively decided for resolving deviations from below −10% to above +10% in steps of 2.5%. This selection was based on observations of tree dieback following severe drought (Buras et al., 2018), for which corresponding pixels in the year of drought featured deviations between −20 and −5%, and, on average, −10%. Thus, red colors in *PDM* maps relate to deviations for which mortality of individual trees has been observed, which however does not necessarily mean that PDM value in the order of −10% does not necessarily indicate mortality. The threshold of −10% also corresponds well with the steep increase in observed tree-mortality caused by Mountain pine beetles in the Rocky Mountains beyond an NDVI decline of 10% (see Figure 6 in Spruce et al., 2019).

Since *Q* allows for a more systematic color-chart in terms of visualization in comparison to *PDM* for which we subjectively decided on color-chart thresholds, we in our study emphasize on *Q* in the main text while *PDM* is shown in the Supplementary Information. Nonetheless, the two metrics can be considered equally meaningful for interpreting variations in forest greenness.

To allow the public to retrieve information based on the EFCM framework, a web-based interactive application has been established at http://interaktiv.waldzustandsmonitor.de/. This Forest Condition Monitor allows for viewing *Q* and *PDM* for the entire European Union for any given date over the monitoring period (2001-now). Users can interactively select which product (*Q* and *PDM*) to display for two different dates, allowing for comparison (e.g., 2003 vs. 2018 as in Buras et al., 2020). *Q* and *PDM* are visualized as colored maps and histograms which allow for quantifying the relative spatial share of specific values of *Q* and *PDM* for the selected dates (the breaks used for the coloration are mentioned above). The underlying data can be downloaded as geo-tiffs. The code underlying the EFCM-viewer has been programmed within the “R-Shiny” environment (Chang et al., 2021) and is provided in the Supplementary Material of this article. Due to high computational costs, the monitored period of the EFCM viewer will be updated twice a year, i.e., at peak season (end of July) as well as at the end of the year.

### Performance Examples

To exemplify the performance of *Q* and *PDM* under different environmental settings, we first depict the temporal development of peak season NDVI (i.e., end of July, represented by DOY 209) from 2001 to 2020 (section Q and PDM From 2001 to 2020). Secondly, we visualize the performance of specific DOY *Q* or *PDM* for selected regions that represent the onset of the growing season, i.e., land-surface phenology (section Land Surface Phenology), forest condition under extreme drought (section Drought), forest condition succeeding late-frost (section Late-frost), forest condition under water-logged soils (section Water Stress), forest condition following an ice storm (section Ice Storm), and forest condition succeeding windthrow (section Windthrow). The specific selection of locations for these examples was based on the knowledge of extraordinary events by the first author of this article.

For the phenology example, we emphasized on Germany and downloaded phenological observations of budburst in beech, which together with pedunculate and sessile oak represents the most abundant deciduous tree species in Germany. Data were obtained from the German Meteorological Service (DWD) for the period 1951–2020. For each year, we computed the mean DOY of budburst over all stations as a representation for the Germany-wide onset of the growing season. We depict *Q* and *PDM* representative of the first of May for the total area of Germany of the year with the earliest onset (2014) and in addition compute a linear regression between mean *Q* and *PDM* and mean budburst (all averaged over Germany) for the common overlap period, i.e., 2001–2020.

In terms of visualizing forest condition under drought, we selected the extreme drought of 2015 which resulted in increased die-back rates of Scots pine in Franconia, Southern Germany (Buras et al., 2018). Here, we depict *Q* and *PDM* for the study region investigated in Buras et al. (2018) for the first of May 2015 (i.e., before the drought), the 13th of August, 2015 (peak of drought), and the first of May 2016 (after the drought). Based on a tree-species cover map (Brus et al., 2012), we extracted *Q* and *PDM* values that corresponded to pixels dominated by Scots pine (relative share per pixel > 70%) and compared those between the three dates visually and using pairwise Wilcoxon rank sum test.

For the late-frost example, we focused on Bavaria in 2011 where a major late-frost event on May 4th (DOY 124) caused foliage damage in beech-trees over a large area (Menzel et al., 2015; Príncipe et al., 2017). We showed maps of *Q* and *PDM* for DOYS 121 and 153 to represent NDVI prior to the event and 4–5 weeks after the event when affected beech trees were about to rebuild their canopy (Menzel et al., 2015). Besides these two time-slices, we also analyze the behavior of *Q* and *PDM* over several time-slices, beginning at DOY 105 and ending at DOY 217, when beech trees presumably had rebuilt their full canopy (Menzel et al., 2015). Thereby, we emphasized on pixels for which beech cover was larger than 45% according to Brus et al. (2012). The selection of 45% as threshold for beech dominance was a necessary compromise, since only few pixels in Bavaria feature beech cover above 50%. Using 45% as threshold left us with 4,938 pixels for the analyses. We then distinguished between beech-pixels which were likely to have experienced frost on May 4th and those which were unlikely to have experienced frost. Frost's likelihood was indicated by thin-plate spline interpolated daily minimum temperature data from climate stations of the German Meteorological service using a digital elevation model at 30 m resolution (SRTM30, Becker et al., 2009) which was remapped to MODIS resolution. The threshold for frost occurrence was set to −1°C to account for the imprecision of the interpolated data whose difference to absolute observations featured a standard deviation of 0.5. Thus, for pixels below −1°C the likelihood of actually experiencing frost on that day is roughly 97.5 %. This left us with 4,708 and 230 beech dominated pixels that were likely or unlikely, respectively, to have experienced late-frost on May 4, 2011.

To exemplify the performance of *Q* and *PDM* under water-stress, we focused on the Darß-Zingst-peninsula in Northern Germany, which is characterized by forests growing on formerly drained and recently rewetted coastal mires. Due to the establishment of the national park, “Vorpommersche Boddenlandschaft,” in 1990, the drainage of mires was gradually stopped over the last decades, wherefore the extremely wet summer of 2011 resulted in waterlogging in large parts of the national park over a period of several months. As a consequence, a remarkable number of trees, mostly alder (*Alnus glutinosa*) and birch (*Betula pubescens*) growing on the formerly drained mires, suffered from water stress and eventually died. To examine how the EFCM mirrors such events, we extracted peak season (DOY 209) *Q* and *PDM* for the years 2010 through 2013 and mapped those for the peninsula. In addition, we extracted *Q* and *PDM* for the two areas that had featured the most remarkable die-back (“Neudarß” and “Sundische Wiesen”) for each time step and compared time steps to each other visually along with using pairwise Wilcoxon rank sum test. To visualize how extraordinary precipitation sums were in 2011, we used climate station data from the German Meteorological Service (DWD) of the closest station with a continuous precipitation record since 1970, i.e., “Groß Lüsewitz” ~40 km south of the peninsula.

For the ice storm example, we focused on the extreme ice storm that hit Slovenia in February 2014 (Roženbergar et al., 2020). Here, we compared the temporal development of *Q* and *PDM* from DOY 97 to DOY 289 (i.e., most of the local growing season) in 2005 for areas that were affected or unaffected by the ice storm, respectively. To identify the corresponding areas, we digitized and georeferenced the map shown in Roženbergar et al. (2020) and applied the resulting shapefile to extract *Q* and *PDM* for the corresponding EFCM maps and categorized the values as either affected or unaffected. Extracted values were statistically compared for each DOY according to the two categories using Wilcoxon rank-sum test.

Regarding the performance of EFCM following windthrow, we focused on the extreme winter storm “Gudrun” which resulted in extraordinary windthrow in southern Sweden in January 2005 (Valinger et al., 2014). For this, we extracted end of June *Q* and *PDM* values for 2005 and grouped them according to maximum wind gust speed, which was downloaded from the Extreme Wind Storms Catalog^3^. We defined groups by subtle storm impact (maximum wind gust <20 m/s, i.e., <9 Beaufort) and then sequentially increasing storm impact in steps of 5 m/s up to wind gusts stronger than 45 m/s. The resulting groups were compared statistically using a one-sided Wilcoxon rank-sum test comparing subtle storm impacts (gust speed <20 m/s) with the remaining groups under the assumption that *Q* and *PDM* should decrease with increasing maximum wind gust speed.

All analyses were computed in R (version 4.0.3, R foundation for statistical computing in Vienna, 2021) extended for the packages raster (Hijmans, 2017), rgdal (Keitt et al., 2011), fields (Nychka et al., 2017), and shiny (Chang et al., 2021).

## Results

### *Q* and *PDM* From 2001 to 2020

**Figure 1:**
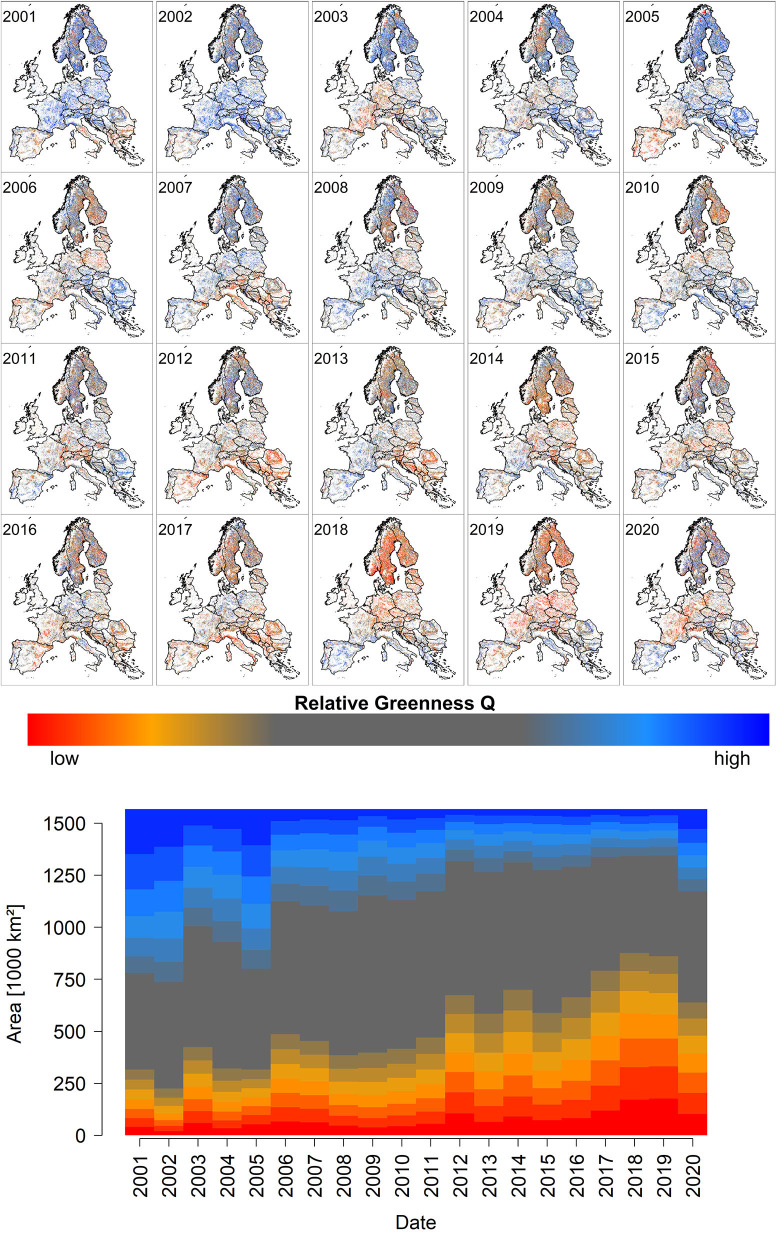
The upper small panels depict the spatiotemporal development of peak season quantiles *Q* over the observational period from 2001 to 2020 (for an animated version with higher resolution please see Supplementary Video 1). The lower stacked barplot depicts the spatial share of corresponding quantiles. The value of *Q* increases from dark red over orange, gray, to blue shades. Supplementary Figure 1 depicts similar information for *PDM*. A video depicting the spatio-temporal variation of *PDM* is shown in the Supplementary Video 2).

End of July EFCM quantiles *Q* and proportional deviations from the median *PDM* show clear trends over the study period from 2001 until 2020 (Figure 1, Supplementary Figure 1). Specifically, the amount of low quantiles and negative deviations (red colors in Figure 1, Supplementary Figure 1) feature an increasing share, which peaks in the years 2018 and 2019. Considering spatiotemporal variations (see Supplementary Videos 1, 2), the well-known major drought events of 2003 (central and southern Europe), 2006 (Baltic states), 2012 (south-eastern Europe), 2015 (central and southern Europe), 2017 (Mediterranean region), 2018 (central and northern Europe), and 2019 (southwestern, central, and northern Europe) are all reflected by regionally low *Q* and *PDM* values (compare Figure 1, Supplementary Figure 1 with Supplementary Figure 2). However, not all the observed extreme values (high and low) are related to drought, as will be shown in Sections Land Surface Phenology, Late-frost, Water Stress, Ice Storm, and Windthrow.

### Land Surface Phenology

**Figure 2:**
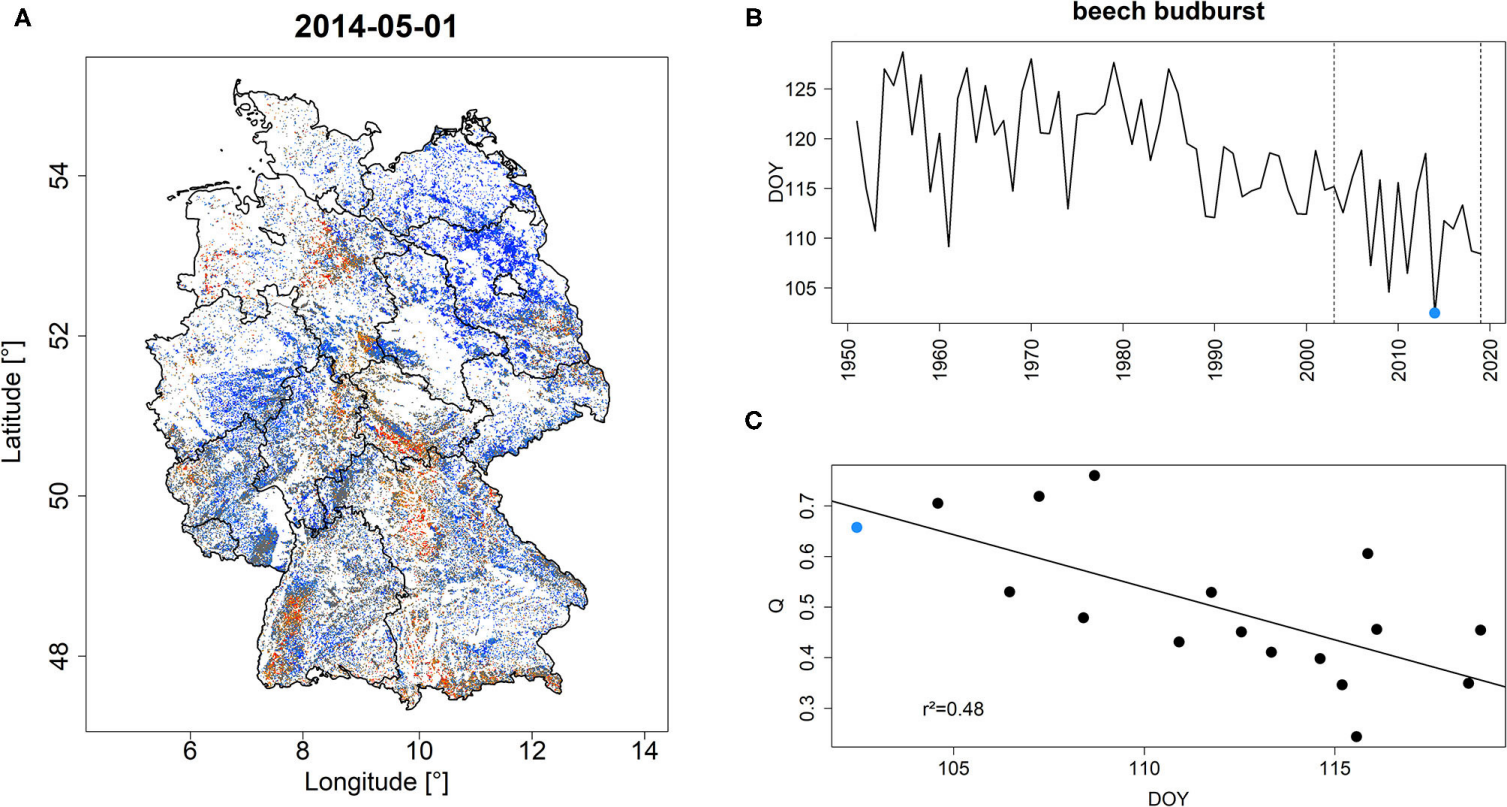
*Q*-values in Germany for May 1, 2014 **(A)** which represents the year with the earliest bud-burst of beech across Germany for the period 1950–2020 **(B)**. *Q* features a significant negative relationship with the average DOY of beech budburst **(C)**. Vertical dashed lines in **(B)** refer to the common overlap period of beech phenology and monitoring period. The blue dot indicates the year 2014 which is depicted in **(A)**. *r*^2^ in **(C)** refers to the explained variance of the depicted regression line. A similar figure but for *PDM* is shown in Supplementary Figure 3.

Early season quantiles featured a significant negative relationship with forest phenology (Figure 2). For instance, the year 2014 which featured the earliest observed budburst of beech across Germany in the period 1950–2020 (blue dot in Figure 2B) was characterized by a high share of high *Q* and *PDM* values (Figure 2A, Supplementary Figure 3A). Over the common overlap period, mean DOY of beech budburst explained 48 and 50% of variance (*p* < 0.001) of *Q* and *PDM* on May 1st, respectively (Figure 2C, Supplementary Figure 3C).

### Drought

**Figure 3:**
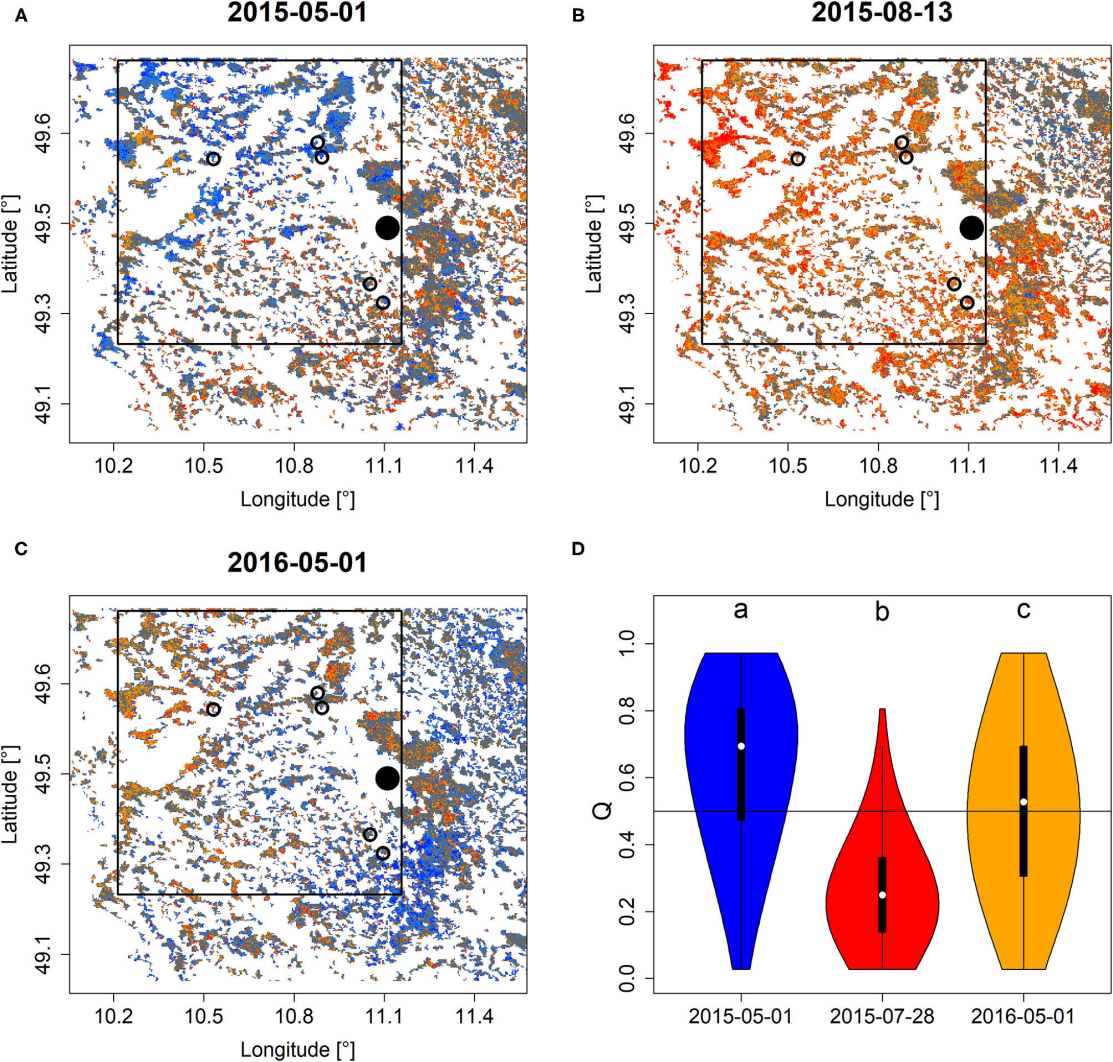
*Q* mapped for the area surrounding Nuremberg (black dot) before **(A)**, during **(B)**, and after **(C)** the extreme drought of 2015. Circles refer to study sites investigated in Buras et al. (2018), in which tree die-back was observed early in 2016. The black rectangle roughly refers to the study area of Buras et al. (2018) for which *Q* was extracted to generate **(D)** which depicts violin plots of *Q*-values of the three corresponding dates of pixels within this rectangle that feature Scots pine cover of more than 70 percent. Minor case letters in **(D)** refer to group-assignments according to Wilcoxon rank-sum test. A similar figure but for *PDM* is shown in Supplementary Figure 4.

Comparing *Q* and *PDM* before and after the extreme drought of 2015 in Franconia, Northern Bavaria indicated a clear and negative response of forests to drought. Particularly during the peak of drought in August 2015 (Figure 3B, Supplementary Figure 4B), large parts of the study area featured extraordinarily low *Q* and *PDM* and overall lower in comparison to the onset of the growing season 2015 (Figures 3A,D, Supplementary Figures 4A,D). Also, at the onset of the growing season of 2016, *Q* and *PDM* featured significantly lower locations (i.e., the non-parametric mean that is tested for in Wilcoxon rank-sum test) if compared to before the drought (Figures 3C,D, Supplementary Figures 4C,D).

### Late-Frost

**Figure 4:**
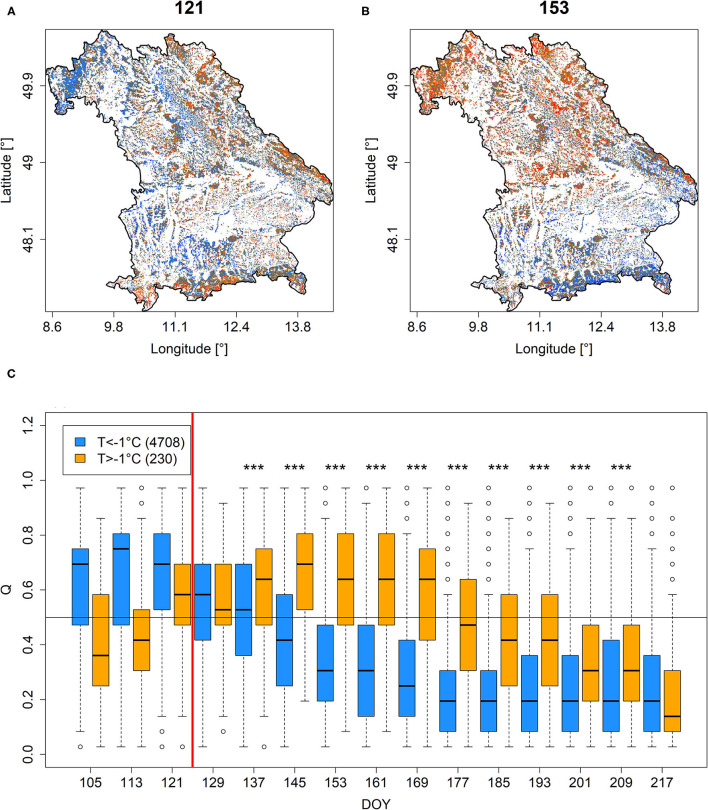
*Q* for DOYs 121 **(A)** and 153 **(B)** in 2011. **(C)** Times-series depicting *Q*-values extracted for each time step between DOY 105 and DOY 217 for pixels featuring more than 45% beech cover and minimum temperatures at DOY 124 (indicated by vertical red line) below (blue) or above (orange)−1°C (for minimum temperature distributions at DOY 124, see Supplementary Figure 6). ***Stars indicate significant differences (*p* < 0.001). A similar figure but for *PDM* is shown in Supplementary Figure 5.

Comparing *Q* and *PDM* on DOY 121 in 2011 (just before the late-frost event on DOY 124) to *Q* and *PDM* on DOY 153 revealed a much higher share of low *Q* and *PDM* 4 weeks after the late-frost event (Figures 4A vs. 4B, Supplementary Figures 5A vs. 5B), particularly in the region that experienced freezing temperatures at DOY 124 (Supplementary Figure 6). If analyzed over time, pixels representing a high share of beech cover (>45%), featured a specific temporal development of *Q* and *PDM* depending on whether they had experienced minimum temperatures below or above −1°C at DOY 124. For pixels that had experienced below −1°C, *Q* and *PDM* decreased over time, whereas they increased over time for pixels that had experienced higher minimum temperatures (Figure 4C, Supplementary Figure 5C). Differences between these two groups remained significant until DOY 217, i.e., early in August.

### Water Stress

**Figure 5:**
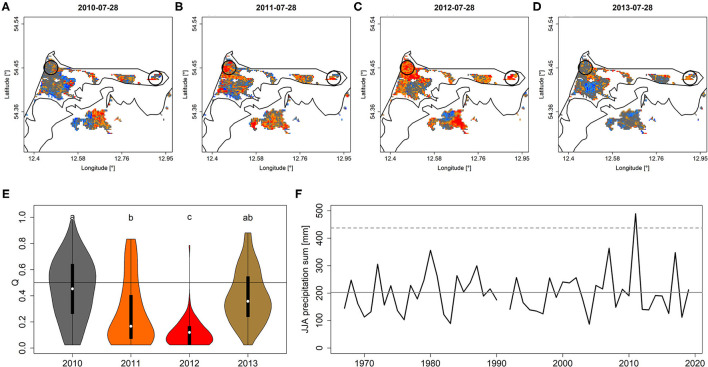
*Q* depicted for peak season (July 28th) for each year from 2010 to 2013 **(A–D)**. Black circles refer to the areas that experienced increased die-back after 2011 (see also Supplementary Figures 8, 9) for which *Q*-values were extracted [shown in **(E)**]. **(F)** June-August precipitation sum from a nearby climate station indicates the year 2011 to exceed the 99% confidence interval (dashed line). Minor case letters in **(E)** indicate group-assignment according to pairwise Wilcoxon rank-sum test. Colors in **(E)** refer to colors of mean quantiles per year. The gray vertical line in **(F)** refers to mean JJA precipitation sum. A similar figure but for *PDM* is shown in Supplementary Figure 7.

The areas on the Darß-Zingst-peninsula that featured tree die-back due to extraordinary precipitation (Figure 5F) and, consequently, flooding during the wet summer of 2011 featured corresponding patterns of *Q* and *PDM*. In particular, peak-season *Q* and *PDM* values of affected areas significantly dropped in 2011 to reach minimum values in 2012 when the die-back occurred and eventually recovered partly in 2013 (Figures 5A–E, Supplementary Figures 7A–E). Aerial photographs of affected areas are shown in Supplementary Figures 8, 9.

### Ice Storm

**Figure 6:**
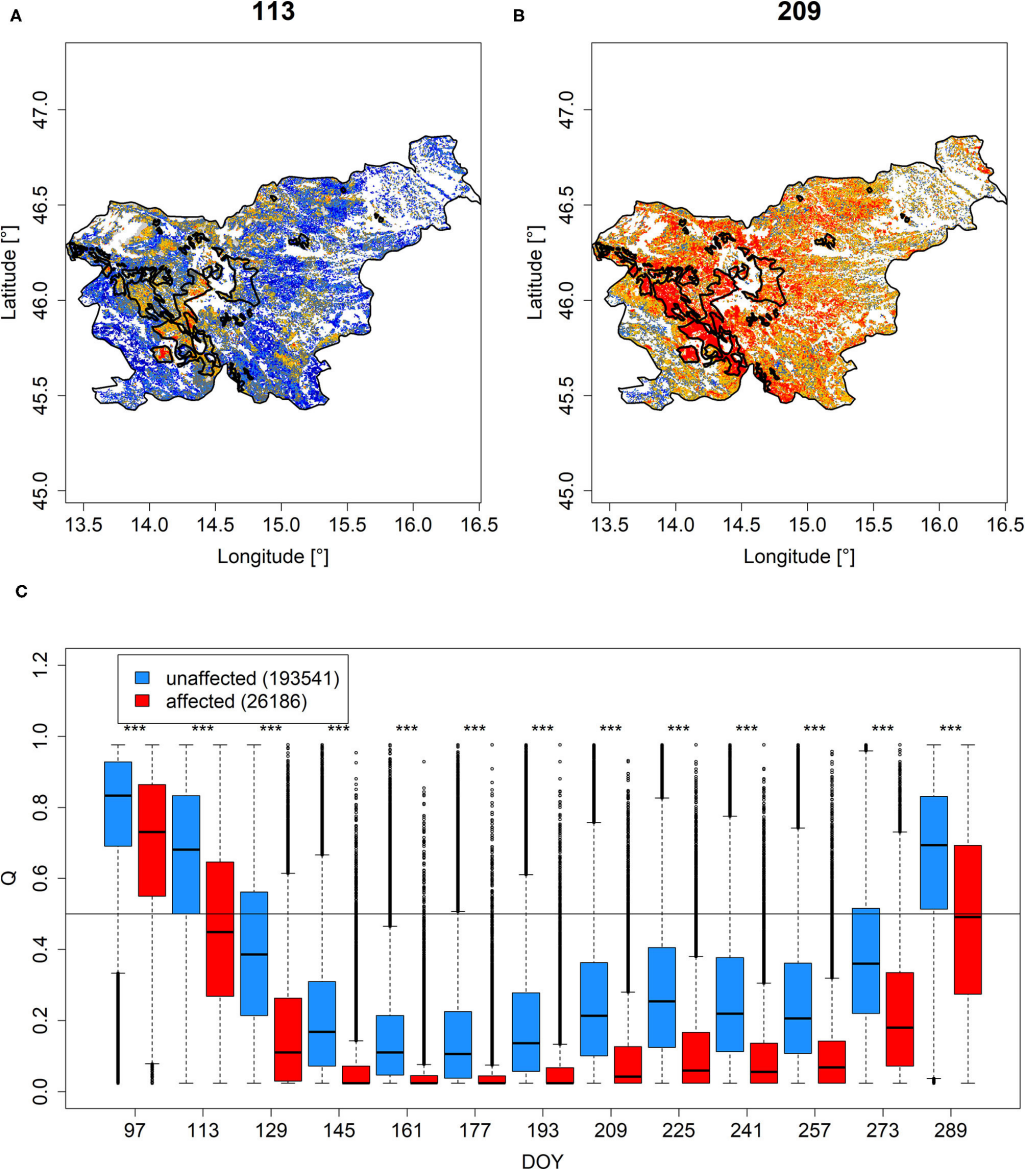
*Q* mapped for Slovenia on DOY 113 **(A)** and 209 **(B)**. The polygons demarcate areas affected by the ice storm according to Roženbergar et al. (2020). Corresponding values grouped according to affectedness from DOY 97 until DOY 289 are shown in **(C)**. The horizontal line in **(C)** demarcates median *Q* values of 0.5. ***Significance stars indicate significant differences between DOY-specific groups according to Wilcoxon rank-sum test. Similar results for *PDM* are shown in Supplementary Figure 10.

The areas in Slovenia that were affected by the ice storm in February 2014 clearly protruded by, on average, lower *Q* and *PDM* throughout the evaluated period (DOY 97-289, Figures 6A,B, Supplementary Figures 10A,B). However, not all areas appeared to have been equally affected, as indicated by remaining high *Q* and *PDM* values in the corresponding group. Nonetheless, time-step specific comparisons revealed statistical significance throughout the whole growing season of 2005, i.e., a significant shift to lower greenness in the affected areas (Figure 6C, Supplementary Figure 10C).

### Windthrow

**Figure 7:**
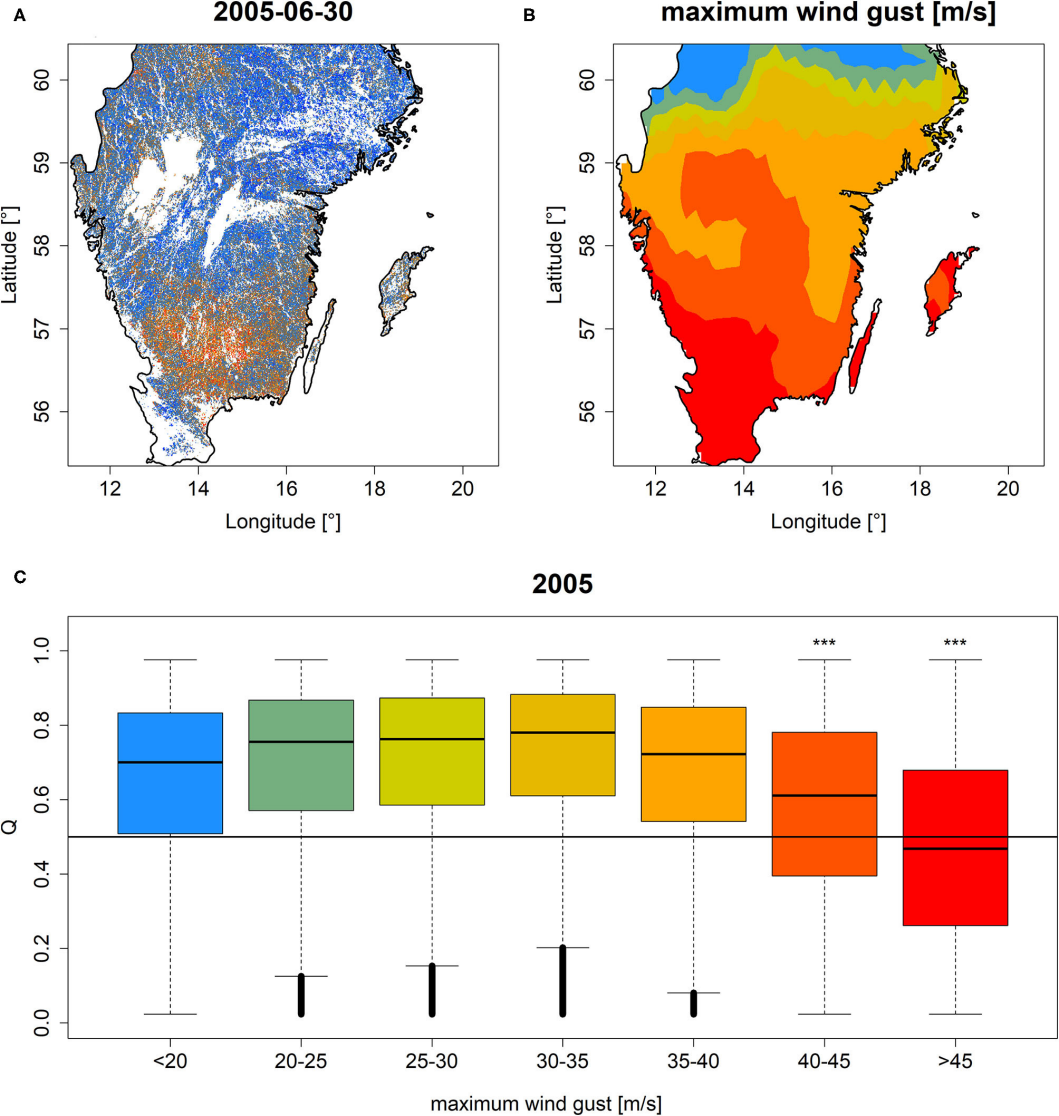
*Q* mapped for southern Sweden on 30th of June, 2005 **(A)** along with maximum wind gust speed during the winter storm “Gudrun” in January 2005 **(B)** and boxplots of *Q* grouped according to different categories of wind gust speed **(C)**. ***Significance stars in **(C)** demarcate a significant (*p* < 0.001) one-sided Wilcoxon rank-sum test between subtle storm impact (gust speed <20 m/s) and the corresponding groups. Colors in **(B,C)** refer to the same wind gust speed categories as indicated on the x-axis of **(C)**. Similar results for *PDM* are shown in Supplementary Figure 11.

The area in Sweden which was hit by extreme wind gusts during the winter storm “Gudrun” in January 2005 featured comparably lower *Q* and *PDM* relative to regions with less extreme wind gusts (Figures 7A,B, Supplementary Figures 11A,B). For wind gusts stronger than 40 m/s, a significantly negative effect on *Q* and *PDM* distributions was observed which was most pronounced for wind gusts stronger than 45 m/s (Figure 7C, Supplementary Figure 11C, Wilcoxon rank-sum test *p* < 0.001 for both *Q* and *PDM*).

## Discussion

### Using MODIS NDVI to Monitor European Forests

The EFCM relies on Terra MODIS NDVI to map the condition of European forests across space and time. The decision to use Terra MODIS NDVI for monitoring European forests is based on the fact that it is provided at a relatively high spatial resolution (231.25 × 231.25 m) with regularly obtained images (in theory daily images which are used to obtain 16-day maximum value composites) that cover a comparably long reference period (2001–2020). Particularly, the regular imaging of land-surface reflectance over long periods renders an important aspect of forest condition monitoring, allowing for tracing changes in greenness in near real-time and relating those to a relatively long monitoring period. While the spatial resolution is superseded by corresponding sensors of the LANDSAT (30 × 30 m) and Sentinel missions (10 × 10 m) by an order of magnitude, these high-resolution images do not provide data at equal temporal resolution. Despite a revisit frequency of 5 days at the equator for Sentinel 2, pixel quality frequently suffers from cloud coverage, both of which, in combination, lead to comparably longer gaps and thus higher uncertainty in corresponding time series. Regarding the reference period, LANDSAT satellites would allow extending the period of Sentinel 2 (beginning in 2015) further back in time however with the same drawback of comparably larger data gaps in comparison to MODIS. Nevertheless, we intend to extend the product portfolio of the EFCM by high-resolution sensors in the future allowing for a more detailed picture of European forest condition.

Regarding the choice of the product, we preferred NDVI over other available vegetation indices that have been used to quantify forest condition in the context of climate change. Among candidate vegetation indices are the solar induced chlorophyll fluorescence (SIF) and the enhanced vegetation index (EVI). While SIF was shown to provide a more precise proxy of GPP in comparison to NDVI (Liu et al., 2018; Shekhar et al., 2020), it suffers from relatively coarse spatiotemporal resolution. SIF observations are usually obtained at irregular frequencies with relatively long return periods for a given location and at the same time typically feature relatively coarse spatial resolutions of more than 1 km^2^ which would not allow for forest monitoring on the aspired scale of individual forest stands. For instance, the NASA Orbiting Carbon Observatory provides SIF at a comparably high resolution (1.3 × 2.25 km) which, however, is insufficient to adequately resolve the heterogeneous landscape patterns in Central and Southern Europe. As a consequence, most of the acquired pixels show a mixed signal which makes it difficult to relate observed fluctuations to actual forest condition. Existing model-based gridded SIF products are able to circumvent the problem of irregular spatiotemporal sampling (Zhang et al., 2018), yet they suffer from a too coarse resolution of 0.05°, i.e., roughly 5 × 5 km in Central Europe. In case SIF-products become available at sufficiently high spatiotemporal resolution, extending the EFCM for valuable additional products will be considered. In contrast to SIF, EVI is also available from the MODIS mission and thus, at a similar spatio-temporal resolution as NDVI. Theoretically, the EFCM could therefore also be based on EVI, but we decided for NDVI since both metrics showed similar patterns for the most prominent European drought events, i.e., 2003 and 2018 (Buras et al., 2020) and NDVI, in comparison to EVI, has been used more frequently in the past for monitoring forest decline and die-back (Kogan, 1995; Xu et al., 2011; Anyamba and Tucker, 2012; Orth et al., 2016; Buras et al., 2018; Rogers et al., 2018; Liu et al., 2019; Brun et al., 2020; Rita et al., 2020) thus allowing for better comparability to existing studies. For the future, we intend to extend the product portfolio of the EFCM by additional vegetation indices such as EVI and SIF to provide a more complete picture of European forest condition. In this context, the next step forward would be to implement early-warning indicators which require integrating information over several time-steps based on statistical properties of NDVI time-series (Liu et al., 2019) along with an extensive testing and validation.

The overall development over the monitoring period of 2001–2020 (section Q and PDM From 2001 to 2020) and more specific case studies (sections Land Surface Phenology, Drought, Late-Frost, Water Stress, Ice Storm, and Windthrow) support the use of NDVI quantiles *Q* and proportional deviations from the median *PDM* for monitoring forest condition. Across Europe, the temporal development of *Q* and *PDM* indicates an increasing forest decline over the last two decades which is in line with the general observation that forests are becoming increasingly stressed in course of climate change (Allen et al., 2010, 2015; Zang et al., 2014; Cailleret et al., 2017; Buras et al., 2018, 2020; Bose et al., 2020; Senf et al., 2020; Shekhar et al., 2020). In comparing clusters of low *Q* and *PDM* (Figure 1, Supplementary Figure 1) with water availability indices, such as the standardized climatic water balance (Supplementary Figure 2), clear similarities of spatial patterns were observed. Finally, spatial patterns of low *Q* and *PDM* mirror reports of well-known major drought events in 2003, 2006, 2012, 2015, 2017, and 2018 (Spinoni et al., 2015; Cindrić et al., 2016; Buras et al., 2018, 2020; Rita et al., 2020; Schuldt et al., 2020). This finding underlines the overarching importance of drought-stress for tree performance and, consequently, mortality. However, as indicated by five of the six presented case studies, extraordinary *Q* and *PDM* values may also be caused by other extraordinary environmental conditions related to an early start of the growing season (section Land Surface Phenology), late-frost (section Late-Frost), mortality on water-logged soils (section Water Stress), mortality following an ice storm (section Ice Storm), and windthrow (section Windthrow). Thus, variations in *Q* and *PDM* always must be interpreted in a comprehensive context, taking further environmental variables such as climate data and phenological observations into consideration.

Besides the EFCM, other national or international remote sensing based (forest) monitoring platforms exist. For instance, on a national level the Sentinel 2 based German “Forest Watch” from the LUP, Potsdam^4^ provides annual updates of the forest condition at a spatial resolution of 10 × 10 m. Thus, while the spatial resolution of Forest Watch is an order of magnitude higher in comparison to the EFCM, it does not allow for resolving different time-steps over the growing season as with the EFCM. Given the comparably shorter reference period of Sentinel 2, Forest Watch only allows for referencing observations against 2017 and, in some regions, 2018 (for details see homepage of Forest Watch). Consequently, the reference period is, to some degree, affected by forest decline (legacies of the drought in 2015, drought in 2018). Moreover, the applied vegetation index is not described in detail, hampering reproducibility and, therefore, comparability with other monitoring tools. Nevertheless, Forest Watch can be considered a complementation to the EFCM with higher spatial, yet lower temporal resolution while interpretations should be undertaken carefully given the rather fixed reference period of one specifically selected year (2017 or 2018). On the international level, the European drought observatory (EDO)^5^ provides various drought-related indices amongst other combined drought indicators and anomalies of the fraction of the absorbed photosynthetically absorbed radiation (fAPAR; Cammalleri et al., 2021), specifically for vegetation. While the availability of several indices is an advantage in comparison to the EFCM, and the monitoring and reference period almost match the EFCM (2002-now), products provided by EDO are of coarser spatial resolution (highest spatial resolution is 500 × 500 m, recurrence period is 10 days). Moreover, since EDO does not specifically monitor forests but vegetated areas in general, direct interpretation of forest condition within a certain area (as provided by the histograms in the EFCM) is not possible. EDO can therefore be considered a valuable source of complementary information which may help interpreting the products provided by the EFCM, particularly in the context of drought-stress. In conclusion, the EFCM renders a novel source of information with regards to European forest monitoring which provides an added value in comparison to existing platforms.

### Evaluating Specific Examples of Forest Monitoring

The observed negative relationships between the two EFCM products and ground-based phenological observations (section Land Surface Phenology) suggest a strong potential to interpret early season *Q* and *PDM* as indicator of land surface phenology. A strong relationship between MODIS NDVI derived land surface phenology and ground observations has been reported earlier (Misra et al., 2016, 2018). However, other factors than the climatically stimulated onset of the growing season may affect early season NDVI, explaining the circumstance that only roughly 50% of the variance in MODIS NDVI was explained by ground observations of beech budburst (see also below). Particularly, years succeeding extreme droughts may feature comparably low NDVI despite an early start of the season, in case the preceding drought has resulted in prolonged forest decline. For instance, after the extreme drought of 2015, a relatively high share of Scots pine forests in Franconia featured partial tree dieback, which to some degree was reflected in lower *Q* and *PDM* values in early 2016 (Figure 3, Supplementary Figure 4). Based on tree-rings and remote sensing observations, drought legacies lasting for 1 year have been frequently reported in the scientific literature (Anderegg et al., 2015; Kannenberg et al., 2018, 2020) which, however, do not always translate directly into reduced forest greenness, e.g., if resources are allocated into canopy repair (greening) instead of secondary growth (Kannenberg et al., 2019, see also section Limitations). Another possible influencing factor is late-frost, which, at least for broadleaved tree-species, may result in delayed greening due to the necessity to rebuilt damaged foliage (section Late-Frost). Also, in the year 2014, a moderate late-frost event stroke parts of southern Germany on April 17th (Supplementary Figure 12), i.e., DOY 107 while the mean budburst of beech took place around DOY 104 (Figure 2C) which may explain some of the red patches observed in Figure 2A. Moreover, negative impacts caused by waterlogged soils, ice storms, or winter storms also result in decreasing forest greenness at the onset of the growing season (sections Water Stress, Ice Storm, and Windthrow). Thus, while it seems likely that high *Q* and *PDM* reflect an early start of season, low values of these metrics do not necessarily indicate a late onset of the growing season. As a consequence, when interpreting early season *Q* and *PDM* phenological observations such as those provided by the Pan European Phenological database, PEP725 (Templ et al., 2018) should be considered as an additional source of information. In case that EFCM observations do not match phenological observations, this potentially indicates a stress situation of corresponding forests which would require further investigation. The close relationship between spring phenology and forest greenness also explains the high share of low *Q* and *PDM* values in spring 2021 (see EFCM viewer) due to a southward location of the polar jetstream over Europe which caused temperatures to be much colder than average (COPERNICUS)^6^ and, consequently, a delay in forest greening.

The impact of the hotter drought of 2015 was also clearly visible in EFCM products (section Drought). While the area of interest in Northern Bavaria featured relatively high *Q* and *PDM* values at the onset of the 2015 growing season, they strongly declined over the summer period due to a strong precipitation deficit (Buras et al., 2018) and only locally recovered early in 2016. Indeed, the regional forest response to 2015 was very heterogeneous, depending on soil conditions, local precipitation events, and stand structure that modify micro-climate. For instance, the observed dieback of Scots pine early in 2016 was most pronounced at south-facing forest edges which, under long-lasting drought, suffer from a warmer and drier micro-climate in comparison to the forest interior (Buras et al., 2018). The resulting heterogeneous response of forests to drought explains the wide distribution of *Q* and *PDM* in early 2016. That is, while some forests had already recovered in 2016, other stands were still negatively affected. In the context of tree mortality, it is important to stress that given the often observed patchiness of tree die-back, the EFCM will not allow for directly identifying individual tree dieback, since the canopy of single trees on average only contribute around 2 permille to the MODIS pixel (10 × 10 m canopy compared to 231.25 × 231.25 m pixel size). However, since low *Q* and *PDM* values likely indicate stressed trees, the likelihood of tree die-back increases with decreasing *Q* and *PDM*. Consequently, low *Q* and *PDM* values during the peak season may inform study site selection if investigating drought-induced tree mortality which then, however, needs confirmation from ground truthing. Since mostly Scots pine was affected by the die-back after 2015, we want to stress the added value of evaluating *Q* and *PDM* in conjunction with local tree-species abundance, since this may allow for more clearly identifying specifically affected tree species. This is also the reason why we only considered pixels with more than 70% Scots pine abundance for this regional example. Tree-species distribution maps such as the one provided by Brus et al. (2012) provide a meaningful source of information within this context. In addition to the example of Scots pine in Franconia, we want to refer to other studies which reported low NDVI for drought-stressed forests which often resulted in increased die-back rates of individual trees (Brun et al., 2020; Buras et al., 2020; Rita et al., 2020; Schuldt et al., 2020).

Besides droughts, late-frost events may have a significant negative impact on NDVI (section Late-Frost). Particularly for broad-leaved tree-species freezing temperatures after leaf unfolding results in loss of foliage and, consequently, a delay in forest phenology and reductions in GPP and, eventually, nonstructural carbohydrates (Menzel et al., 2015; Príncipe et al., 2017; Bascietto et al., 2018; D'Andrea et al., 2019; Vitasse et al., 2019). Here, we could show a differing temporal NDVI development of beech-covered MODIS pixels with contrasting exposure to freezing temperatures in May 2011. Significant effects were observable until the end of July (DOY 209, see Figure 4, Supplementary Figure 5C), i.e., well-beyond the time, it took to re-establish a mature canopy in a heavily affected beech-stand in the Bavarian Forest where beech canopies had reestablished around DOY 178 in 2011 (Menzel et al., 2015). This example indicates how the EFCM may allow for quantifying impacts of late-frost events on a large scale, which then can be combined with ground-based observations as derived from tree-rings (Príncipe et al., 2017; Vitasse et al., 2019), eddy covariance flux measurements (Bascietto et al., 2018), or measurements of soluble sugar concentrations and non-structural carbohydrates (D'Andrea et al., 2019) to gain a deeper understanding of intermediate and midterm tree response. Our late-frost example is in line with Bascietto et al. (2018) who also reported a different seasonal development of satellite borne vegetation indices (in their case enhanced vegetation index, EVI) in dependence of late-frost damage. Yet again, care needs to be taken when interpreting the temporal development of *Q* and *PDM* since it may be affected by other environmental conditions as well. For instance, the ongoing decline of *Q* and *PDM* over the growing season 2011 in Bavaria for all beech-covered pixels independent of frost damage is likely related to the extremely wet and relatively cool summer in that year, which in other regions resulted in tree die-back on water-logged soils (section Water Stress). Also, if trees suffer from legacies of preceding droughts (section Drought), late-frost patterns as observed in 2011, which were not affected by preceding drought, may be obscured. Thus, when interpreting late-frost impacts based on the EFCM, additional information from climate station data and phenological observations on budburst of potentially affected tree species needs to be considered, too. As for the drought example, we want to stress the importance of considering species-specific reactions to late frost. It is obvious that evergreen coniferous tree-species are likely less affected by late-frost in comparison to deciduous tree-species. And even among deciduous tree-species, varying vulnerability to late-frost, in dependence of species-specific budburst and defense mechanisms, is likely (Lenz et al., 2013). Therefore, if studying impacts of late-frost on forest performance, we recommend incorporating information on tree-species abundance for the area under study as exemplified here.

In section Water Stress, we have shown that stand-wide die-back of trees is clearly reflected by the EFCM. In course of the extremely wet summer of 2011, formerly drained and recently rewetted coastal mires on the Darß-Zingst peninsula featured water-logged soils during summer 2011, i.e., at a time when trees growing on these mires usually had experienced comparably lower ground-water tables throughout their lifetime. As a consequence, tree roots suffered from anoxia during a period of active photosynthesis which eventually led to die-back of most trees in the affected areas (see Supplementary Figures 8, 9). The die-back was clearly reflected in *Q* and *PDM* for 2011 and 2012. While in 2013, the reduction in NDVI compared to 2010 partly recovered to values around the median. We explain this relaxation from low NDVI values by the impact of the forest understory, which established after the die-back and consequently raised the NDVI signal where canopies of dead trees had disappeared. In conclusion, while the EFCM is able to resolve the die-back of complete stands efficiently, the replacement of dead trees by successive vegetation will lead to a recovery of NDVI, which however does not indicate an immediate recovery of forests. The same holds true for other causes of large-scale tree die-back, wherefore recovering NDVI values succeeding extremely low values should always be interpreted carefully. At best, ground-based observations or remote sensing products with a higher spatial resolution are considered to obtain more detailed information on the causes of NDVI recovery (Senf et al., 2020). The observation that the EFCM resembles tree die-back is well in line with previous studies, which reported declining NDVI for forests suffering from bark-beetle calamities and/or drought (Rogers et al., 2018; Liu et al., 2019; Spruce et al., 2019).

Also, tree damage and mortality, because of the Slovenian ice storm in February 2014, was well-captured by the EFCM metrics (section Ice Storm). First, patterns of canopy damage were clearly visible when mapping *Q* and *PDM* (Figures 6A,B, Supplementary Figure 10). Comparing unaffected areas with affected areas, both *Q* and *PDM* featured significantly lower values for affected pixels over the whole growing season of 2005 (Figure 7C, Supplementary Figure 10). The observation that some of the presumably affected pixels yet featured relatively high values possibly refers to the specific impact of the ice storm in dependence of tree species and tree size (Roženbergar et al., 2020) but also the varying local impact of the ice storm which was not represented using the digitized shape with a binary classification.

Finally, the windthrow in southern Sweden caused by the extreme winter storm “Gudrun” in February 2005 (Valinger et al., 2014) was spatially well resembled by *Q* and *PDM* (section Windthrow). Particularly, above a maximum wind gust speed of 40 m/s, significantly negative effects on EFCM metrics were observed. Yet, the observation of high values in regions presumably affected by extreme wind gusts was surprising. We hypothesize that this observation is caused by a multitude of factors which affect the critical wind gust speed above which a tree is thrown and variations in local turbulence intensity and wind gust duration which result in heterogeneous, patchy patterns of gust impact that are not represented by interpolated wind gust data (England et al., 2000). Finally, according to Valinger et al. (2014) windthrow in 2005 almost exclusively affected Norway spruce (*Picea abies* L.) wherefore the canopy greenness of other tree species (particularly, broadleaved tree species) likely contributed to the observed heterogeneous signal. To better understand the observed heterogeneous patterns of *Q* and *PDM*, more detailed ground-truthing, for instance, from forest inventories, would be needed. Nevertheless, the windthrow caused by the winter storm in 2005 exemplifies another potential cause of low *Q* and *PDM* which should be taken into consideration when interpreting products from the EFCM.

### Limitations

As already pointed out in section Evaluating Specific Examples of Forest Monitoring, interpretations derived from EFCM products should be interpreted carefully and in conjunction with other environmental information to get a more complete picture of potential underlying causes. One specific limitation of the EFCM is related to the spatial resolution of 231.25 × 231.25 m (roughly 5.3 hectares) which is too coarse to resolve single trees and further results in statistical noise in pixels including non-forest areas. Since various environmental conditions may result in extraordinarily low NDVI, it may be difficult to clearly identify the cause of observed effects. To partly overcome these limitations, we recommend additionally considering high-resolution satellite imagery as, e.g., provided by the LANDSAT and Sentinel missions. Based on their finer spatial resolution, they likely allow for canceling out noise caused by non-forest land cover and in case of Sentinel 2 potentially to backtrack low NDVI values to specific tree groups. Moreover, considering ecological climate parameters as derived from climate station data and phenological observations may help to better understand observations of extraordinary NDVI. As mentioned earlier, we intend to extend the EFCM with additional remote-sensing based vegetation indices from additional sensors, allowing for a more comprehensive picture of forest condition.

We want to stress that EFCM products do not translate directly into forest productivity and vitality. While NDVI and productivity and vitality are linked to some degree, this link may be violated under specific circumstances. For instance, so-called masting events of beech, which result in reduced NDVI due to a high share of beechnuts in the canopy, may lower NDVI while high GPP is maintained. Again, it, therefore, seems mandatory to always interpret EFCM products in the context of other environmental constellations and consider additional measures of productivity such as eddy covariance flux measurements (e.g., Bascietto et al., 2018; Pastorello et al., 2020) and solar induced fluorescence (Liu et al., 2018; Shekhar et al., 2020). In addition, when interpreting *Q* and *PDM* in the context of tree mortality, care needs to be taken. While the likelihood of tree mortality is likely to increase in course of declining canopy greenness (Rogers et al., 2018; Liu et al., 2019; Spruce et al., 2019), trees may yet recover from the causal environmental stressors. Consequently, additional ground truthing is needed to confirm the actual death of trees, which on a larger scale may be derived from satellite-based canopy mortality assessments (Senf et al., 2020).

Within this context, we want to highlight the possible mismatch between measures of secondary growth (e.g., indexed tree-ring width) and canopy greenness as represented by NDVI (Kannenberg et al., 2019). For instance, Kannenberg et al. (2019) observed significantly reduced tree-ring width during drought and in the subsequent year while GPP and NDVI only featured significant reductions during drought but not in the following year. These authors hypothesized this observation to be caused by post-drought photosynthesis upregulation and carbon allocation in favor of canopy repair (recovering NDVI) at the expense of secondary growth (reducing tree-ring width). In another study, the link between NDVI and secondary tree-growth differed between non-drought and drought conditions (Meyer et al., 2020): while tree-ring width was most closely linked with the start of season NDVI under non-drought conditions, peak-season NDVI better reflected secondary growth decline under drought conditions. The link of spring NDVI with tree-growth under normal conditions is probably caused by the overarching effect of spring conditions on secondary growth of trees growing in temperate climates (Buras et al., 2018; Kannenberg et al., 2019), while the link under drought conditions with summer NDVI likely reflects early leaf coloration or senescence which result in reduced ring-widths (Meyer et al., 2020; Schuldt et al., 2020). On the global scale, the relationship between secondary growth and canopy greenness is further modified by tree-species, climate zone, and integration period of the NDVI signal (Vicente-Serrano et al., 2016; Bhuyan et al., 2017). In conclusion, care needs to be taken when translating fluctuations of *Q* and *PDM* into GPP and variations of secondary growth.

Regarding *PDM*, the subjectively chosen steps for the color-charts may be considered a potential weakness. The main reason for adding *PDM* is the complementation of *Q* which does not allow for quantifying the absolute deviation from the long-term median in contrast to *PDM*. Since *PDM* however also requires visualization, we defined the relatively arbitrary thresholds described in section Computation of Central Metrics. While these thresholds are based on forest decline observations made by the first-author after a severe drought in 2015 (Buras et al., 2018), we admit that such thresholds likely are site and tree-species specific. Nevertheless, the chosen lower threshold of −10% is well in line with a sharp increase in tree mortality observed in course of a Mountain pine beetle calamity in the western U.S. (Spruce et al., 2019). Based on the examples shown, the color-chart applied to *PDM* often resulted in a less pronounced impression of forest decline in comparison to *Q*. However, when studying the supplied box and violin-plots, *PDM* generally supported significant results reported for *Q*, although differences again appeared to be less pronounced. This latter impression comes from the fact, that *PDM* in comparison to *Q* does not feature a bounded distribution between 0 and 1, wherefore whiskers of the corresponding plots stretch to more extreme values, thereby affecting the y-axis limits. As a consequence, boxplot comparisons for *PDM* suggested smaller differences in comparison to *Q*, which however was not the case (both metrics are based on the same underlying data). Given these potentially ambiguous effects related to the visualization of *PDM*, we decided to emphasize our performance examples on *Q*. Despite these drawbacks regarding visualization, we consider *PDM* a required complementation to *Q* allowing for quantifying the proportional deviation of forest greenness from the long-term median.

## Conclusion and Outlook

Based on an assessment of the overall development of peak season NDVI quantiles and proportional deviations from the median and six local case-studies, we have shown that the framework behind the European Forest Condition Monitor provides meaningful information on forest condition at a continental and local scale (sections Using MODIS NDVI to Monitor European Forests and Evaluating Specific Examples of Forest Monitoring). To understand the causes of observed effects, we recommend consulting additional sources of environmental information such as climate station data, phenological observations, and additional high-resolution satellite imagery to overcome limitations associated with the relatively coarse spatial resolution (section Limitations). If interpreted in conjunction with additional environmental information, the EFCM allows for identifying hotspots of forest decline across Europe since 2001 and may therefore serve as a decision tool for designing forest vulnerability assessments in the context of extreme environmental conditions. The ecologically meaningful performance of the EFCM supports the underlying methodological approach based on MODIS NDVI. An interactive web application that allows for viewing and downloading corresponding images and data of *Q* and *PDM* for any time-step since 2001 across the entire European Union is available at http://interaktiv.waldzustandsmonitor.de/. Future updates of the EFCM will aim at extending the product portfolio integrating additional vegetation indices (e.g., EVI, SIF) and additional sensors (e.g., Sentinel 2, LANDSAT 8) which in combination provide a better estimate of actual forest condition at a higher spatial resolution. Updates on the further refinement of the EFCM will be posted in the newsfeed of the forest condition monitor homepage at www.waldzustandsmonitor.de.

## Data Availability Statement

Publicly available datasets were analyzed in this study. This data can be found at: https://lpdaacsvc.cr.usgs.gov/appeears.

## Author Contributions

AB invented, conceptualized, and published the forest condition monitor in 2019 and developed the shiny app in 2021. He also designed the research underlying this study, performed all analyses, and drafted the manuscript. AR and CZ provided valuable suggestions on how to improve the visualization of the forest condition monitor and commented on the manuscript draft. All authors contributed to the article and approved the submitted version.

## Funding

This project was funded by the Bavarian Ministry of Science and the Arts in the context of the Bavarian Climate Research Network (BayKliF).

## Conflict of Interest

The authors declare that the research was conducted in the absence of any commercial or financial relationships that could be construed as a potential conflict of interest.

## Publisher's Note

All claims expressed in this article are solely those of the authors and do not necessarily represent those of their affiliated organizations, or those of the publisher, the editors and the reviewers. Any product that may be evaluated in this article, or claim that may be made by its manufacturer, is not guaranteed or endorsed by the publisher.

## Footnotes

^1^AppEEARS Team (2021). Application for Extracting and Exploring Analysis Ready Samples (AppEEARS). NASA EOSDIS Land Processes Distributed Active Archive Center (LP DAAC), USGS/Earth Resources Observation and Science (EROS) Center, Sioux Falls, South Dakota, USA. Available online at: https://lpdaacsvc.cr.usgs.gov/appeears (accessed September 16, 2021).

^2^COPERNICUS Corine land cover: https://land.copernicus.eu/pan-european/corine-land-cover.

^3^Extreme Wind Storm Catalog. Available online at: http://www.europeanwindstorms.org, Copyright Met Office, University of Reading and University of Exeter.

^4^Forest Watch, LUP Potsdam, http://forestwatch.lup-umwelt.de/.

^5^Available online at: https://edo.jrc.ec.europa.eu.

^6^COPERNICUS Report on spring 2021: https://climate.copernicus.eu/spring-2021-europe-was-it-really-so-cold.
