# Supplementary material for: The European Forest Condition Monitor: Using Remotely Sensed Forest Greenness to Identify Hot Spots of Forest Decline
Source: Front Plant Sci. 2021 Dec 1;12:689220. doi: 10.3389/fpls.2021.689220 (PMC8672298; doi:10.3389/fpls.2021.689220)
Supplement: Supplementary file 3 [file Data_Sheet_1.PDF]

Supplementary Material

1. Supplementary Figures

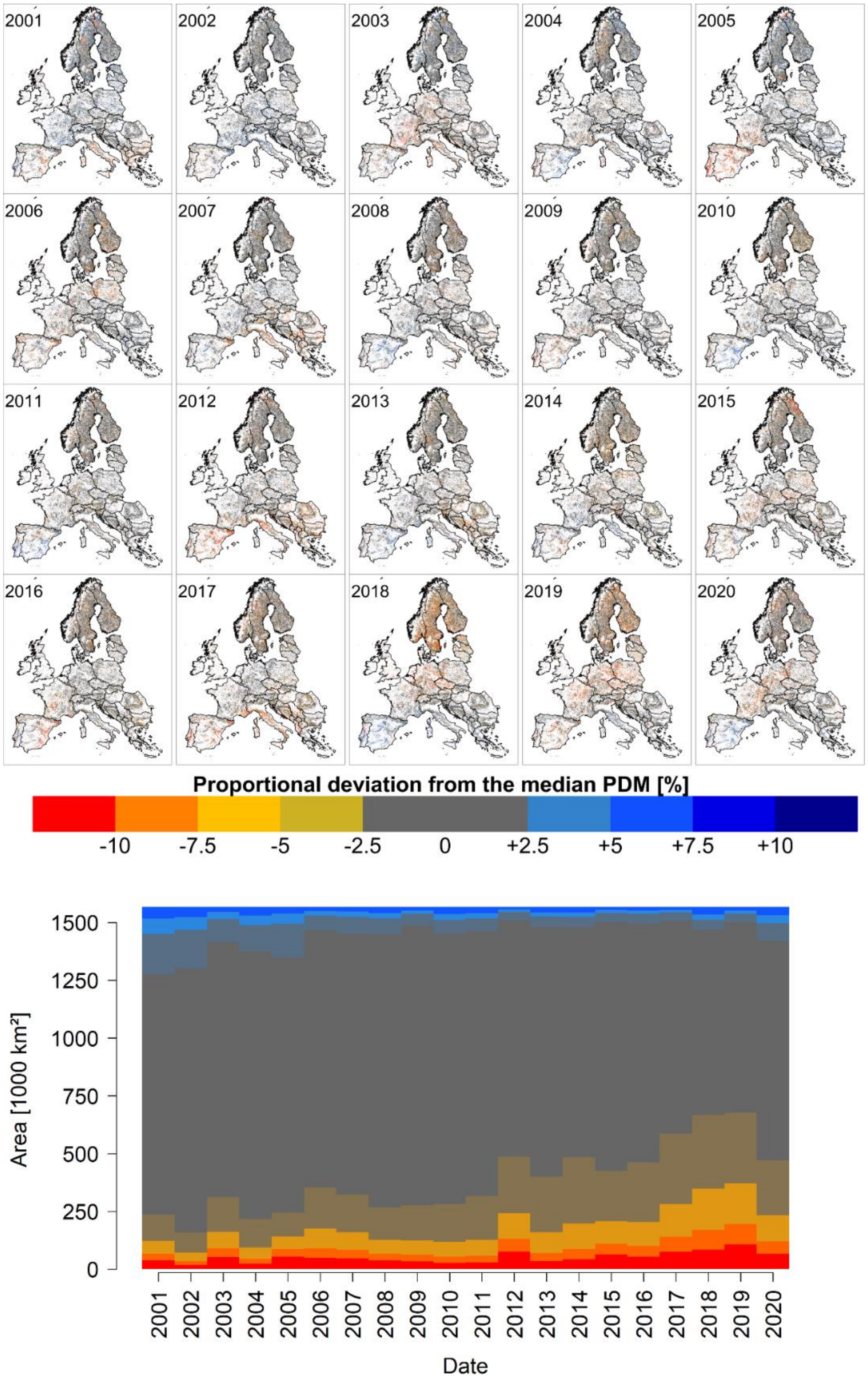

Fig. S1: The upper small panels depict the spatiotemporal development of peak season proportional deviations from the mean, i.e. *PDM* over the observational period from 2001-2020 (for an animated version with higher resolution please see supplementary videos V1 and V2). The lower barplot depicts the spatial share of corresponding *PDM* values. The value of *PDM* increases from dark-red over orange, grey, to blue shades.

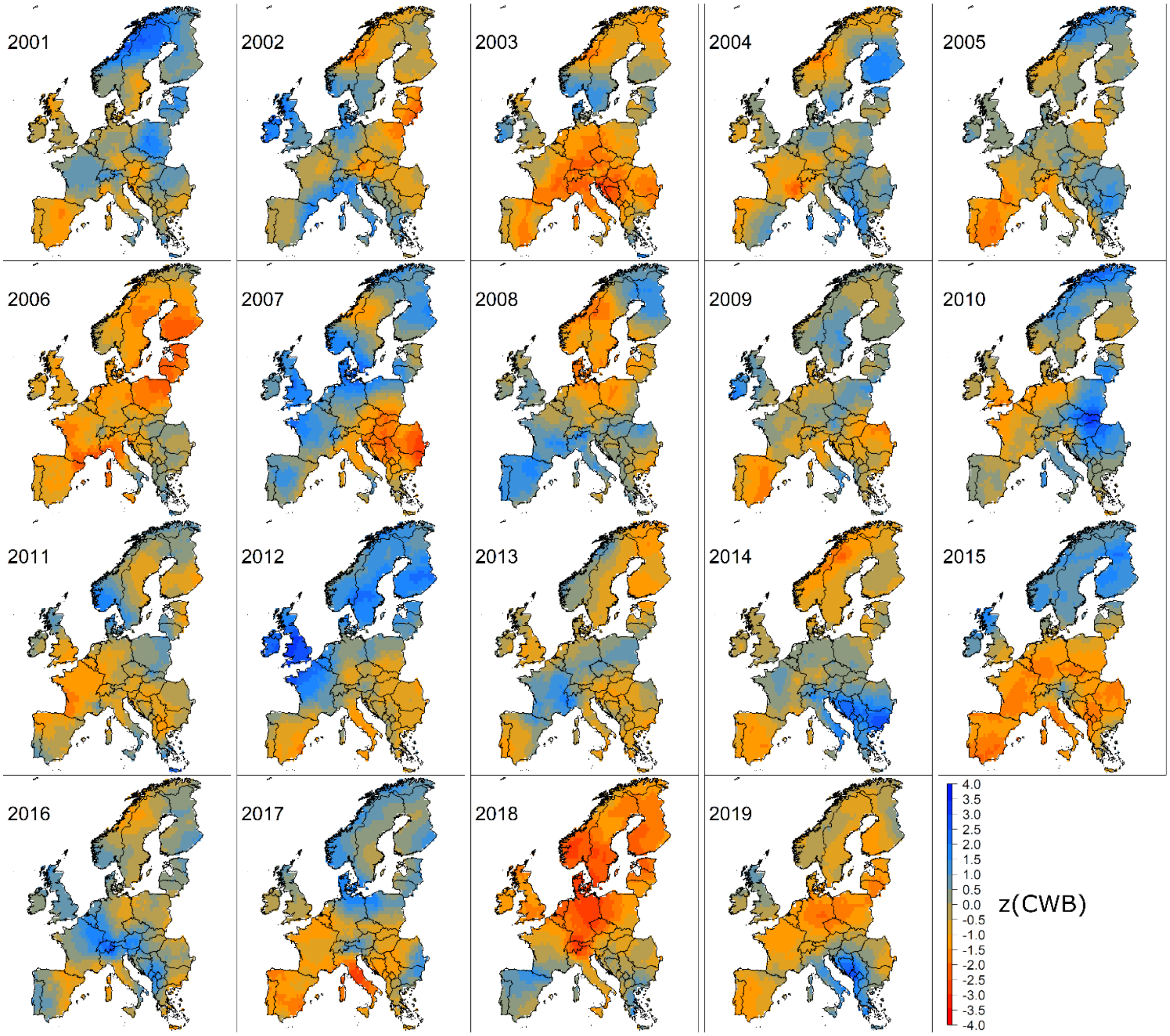

Fig. S2: Standardized climatic water balance z(CWB) integrated over four months for Europe over the period 2001-2019. Red-orange colours indicate negative deviations, while Blue shading refers to positive deviations.

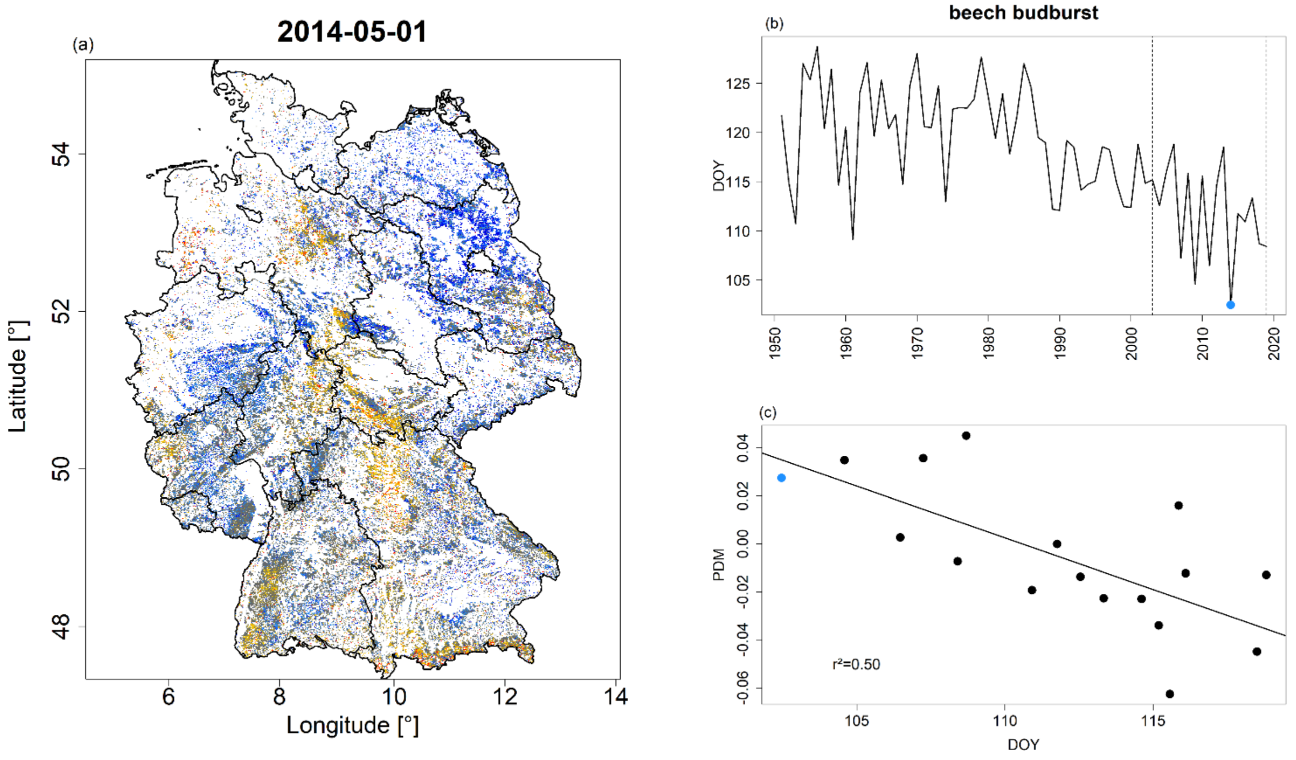

Fig. S3: *PDM*-values in Germany for May 1st 2014 (a) which represents the earliest bud-burst of beech across Germany for the period 1950-2020 (b). *PDM* features a significant negative relationship with the average DOY of beech budburst (c). Vertical dashed lines in (b) refer to the common overlap period of beech phenology and *PDM*. The blue dot indicates the year 2014 which is depicted in (a). r^2 in (c) refers to the explained variance of the depicted regression line.

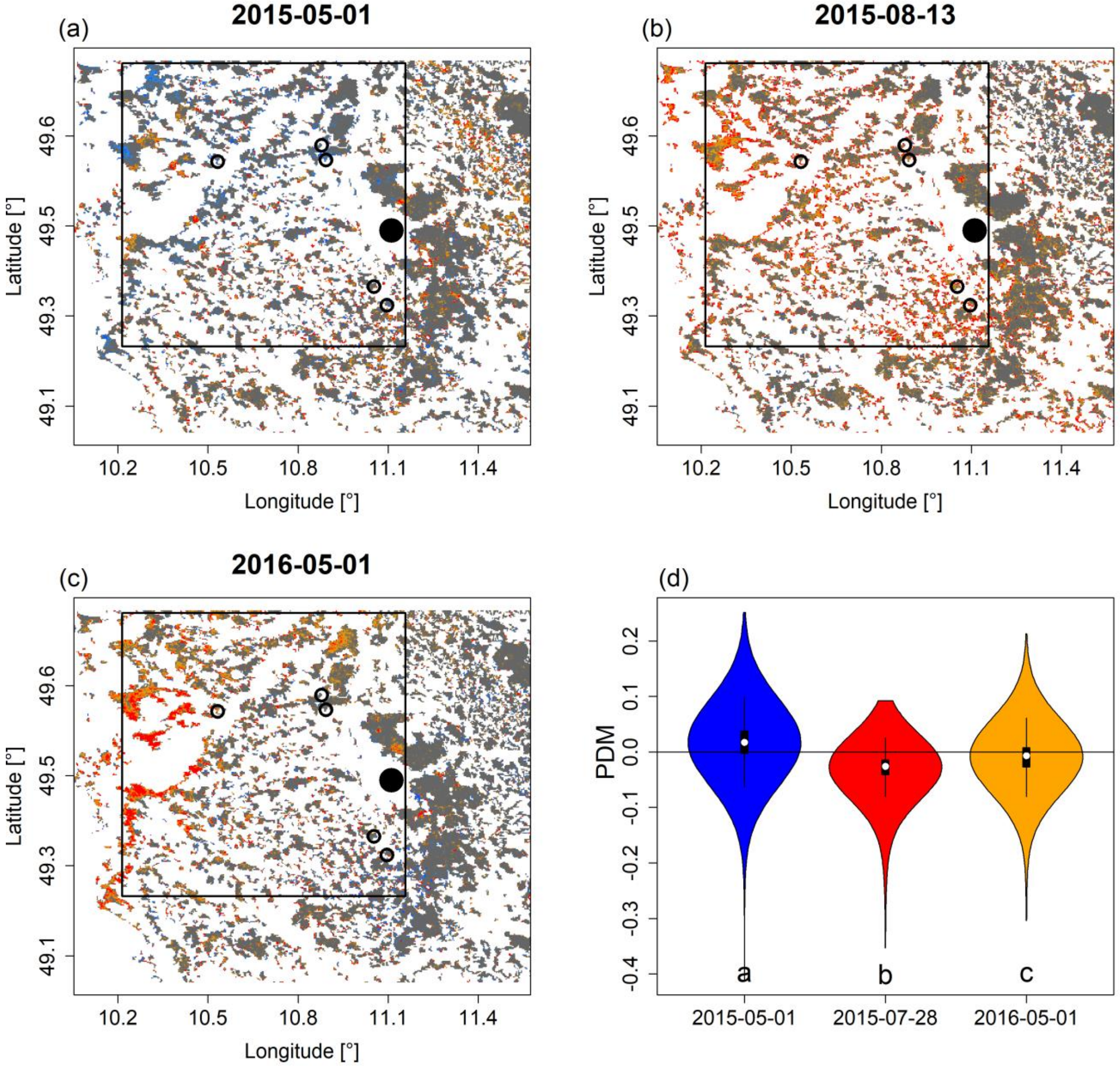

Fig. S4: *PDM* mapped for the area surrounding Nuremberg (black dot) before (a), during (b), and after (c) the extreme drought of 2015. Circles refer to study sites investigated in Buras et al. (2018), in which tree die-back was observed early in 2016. The black rectangle roughly refers to the study area of Buras et al. (2018) for which *PDM* was extracted to generate (d) which depicts violin plots of *PDM*-values of the three corresponding dates of pixels within this rectangle that feature Scots pine cover of more than 70 percent.

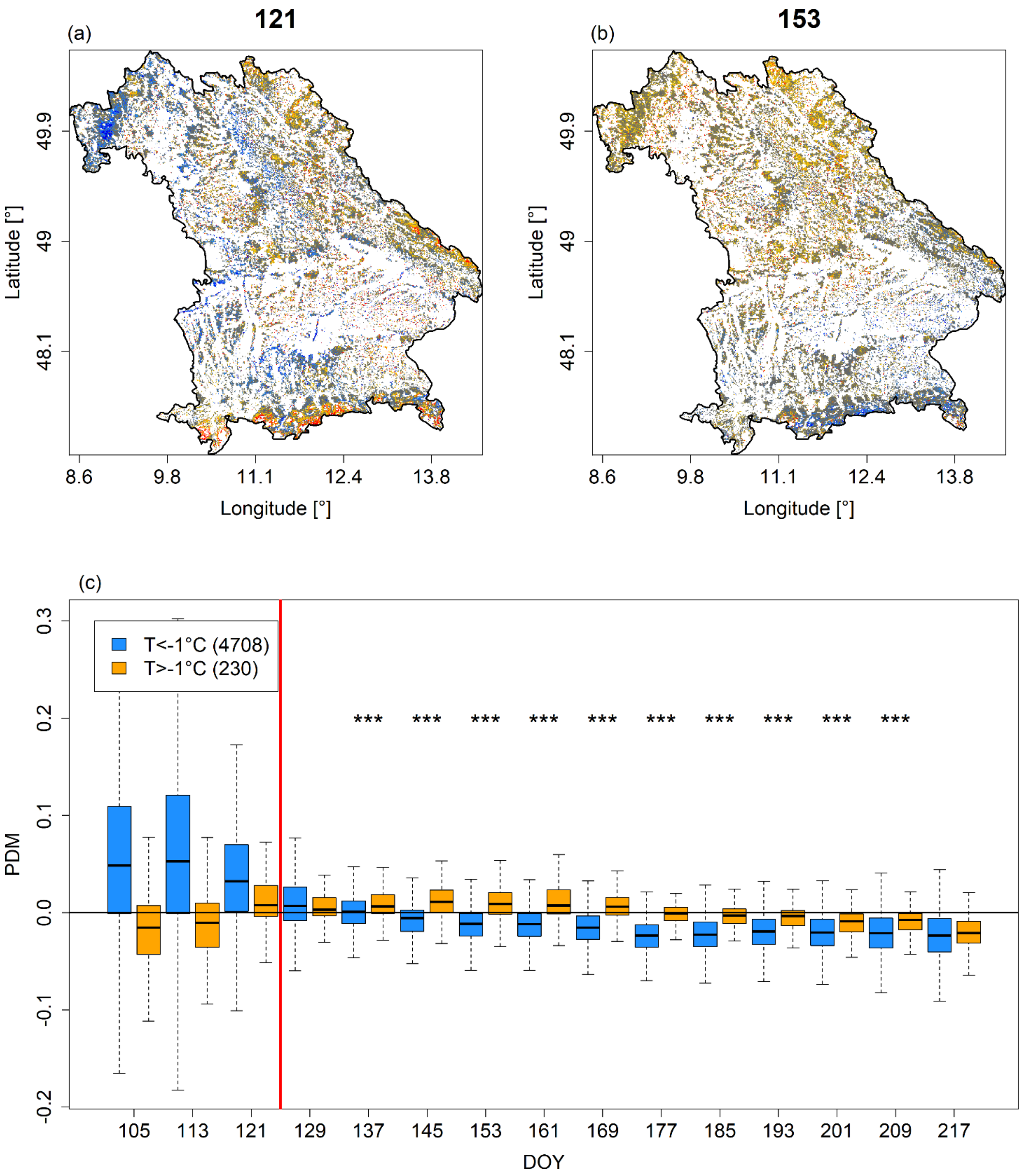

Fig. S5: *PDM* for DOYs 121 (a) and 153 (b) in 2011. Times-series depicting *PDM*-values extracted for each time step between DOY 105 and DOY 177 for pixels featuring more than 45% beech cover and minimum temperatures at DOY 124 (indicated by vertical red line) below (blue) or above (orange) -1°C (for minimum temperature distributions on DOY 124, see Fig. S6).

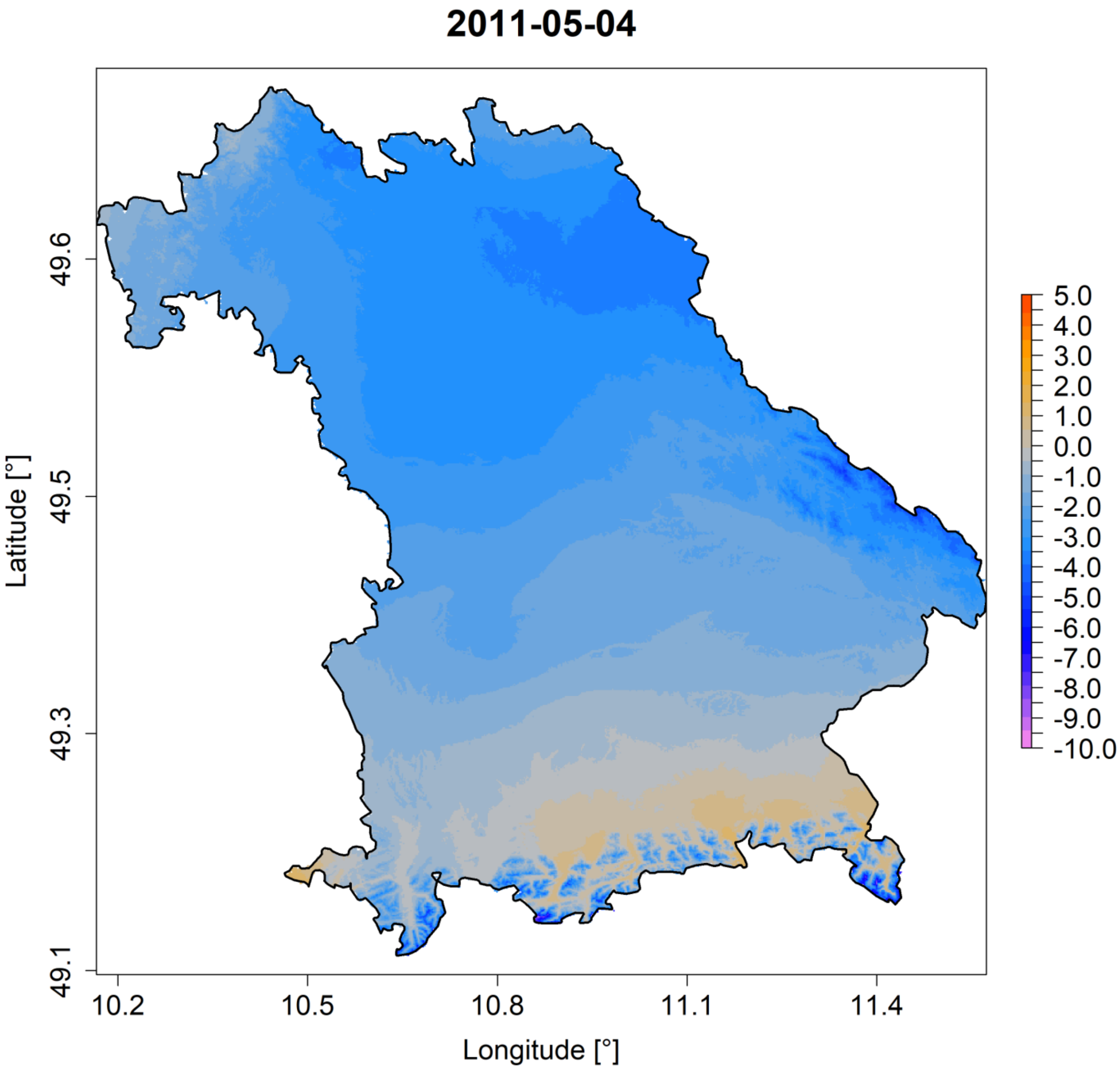

Fig. S6: Thin-plate spline interpolated minimum temperature for May 4th 2011 across Bavaria.

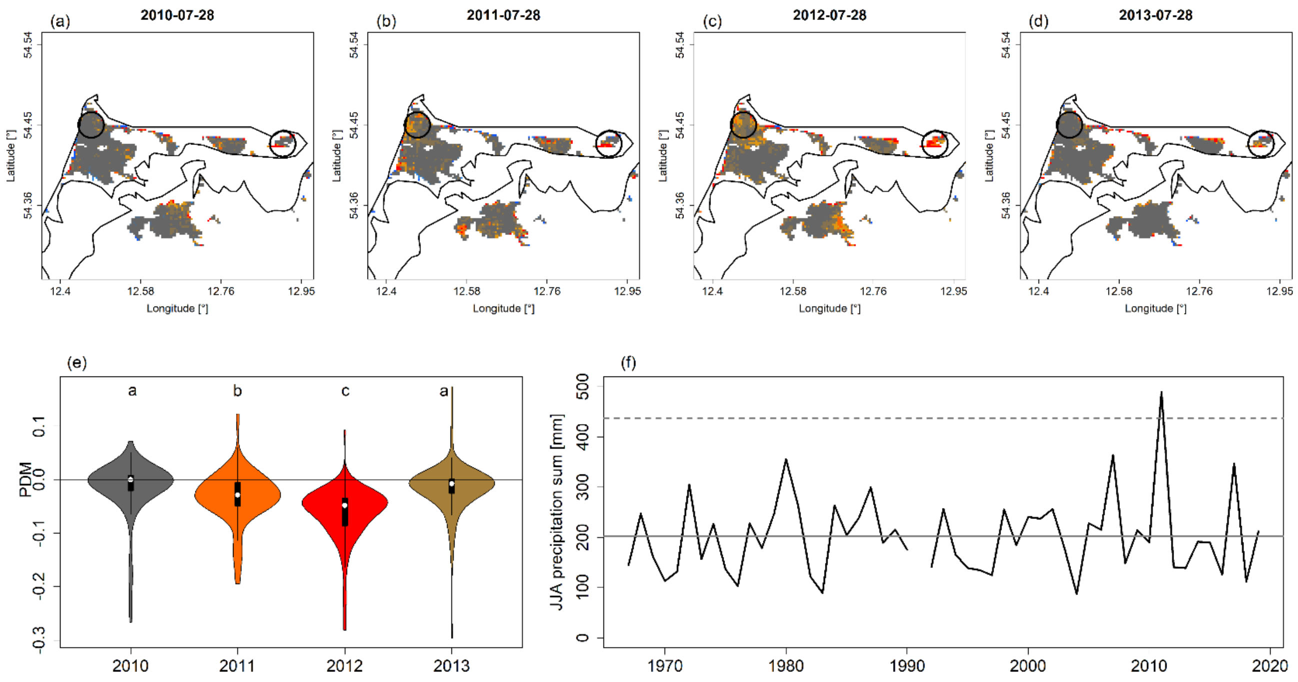

Fig. S7: *PDM* depicted for peak season (July 28th) for each year from 2010 to 2013 (a-d). Black circles refer to the areas that experienced increased die-back after 2011 (see also Figs. S8 and S9) for which *PDM*-values were extracted (e). June-August precipitation sum from a nearby climate station indicates the year 2011 to exceed the 99% confidence interval (dashed line). Minor case letters in (e) indicate grouping according to pairwise Wilcoxon rank-sum test. The grey vertical line in (f) refers to mean JJA precipitation sum.

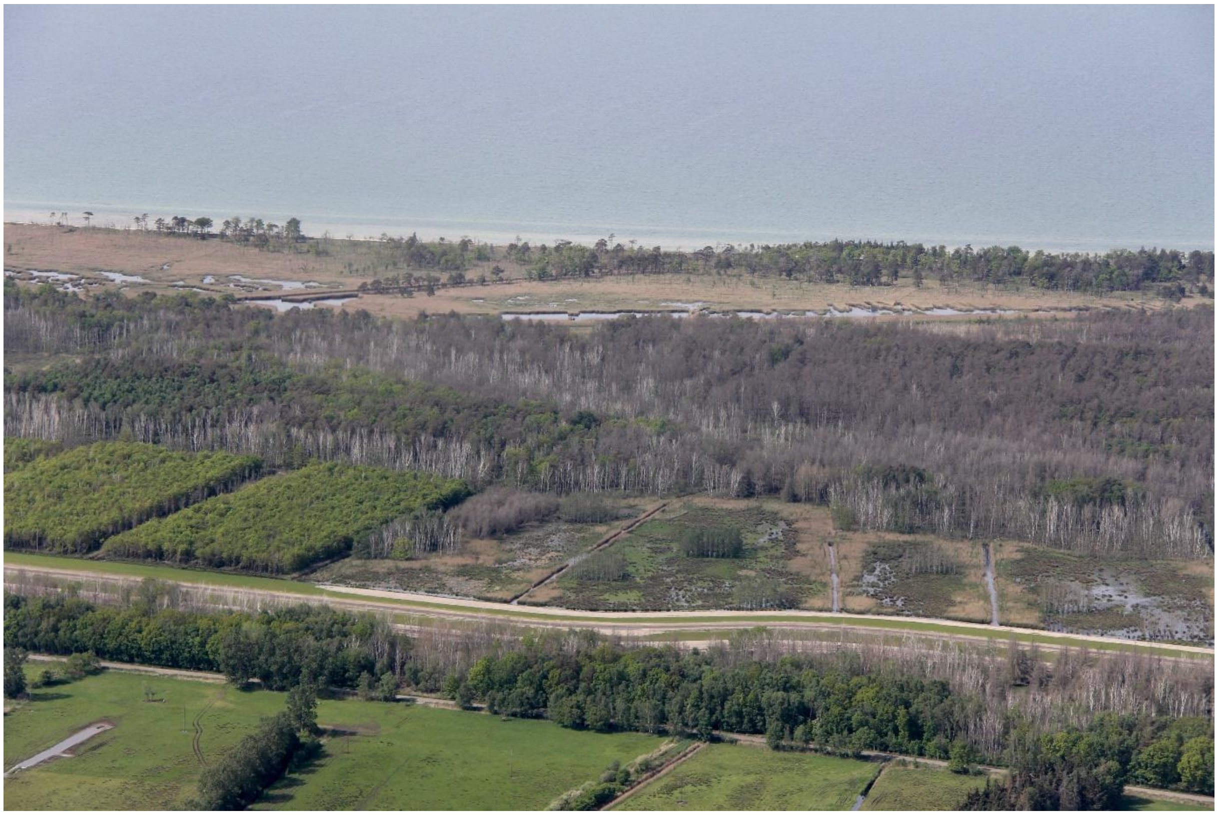

Fig. S8: Aerial photograph of the eastern location indicated in Fig. 5 from Mai 2013. Almost all birches in this region died after the wet summer 2011 due to stagnating water. Image courtesy of Lutz Storm

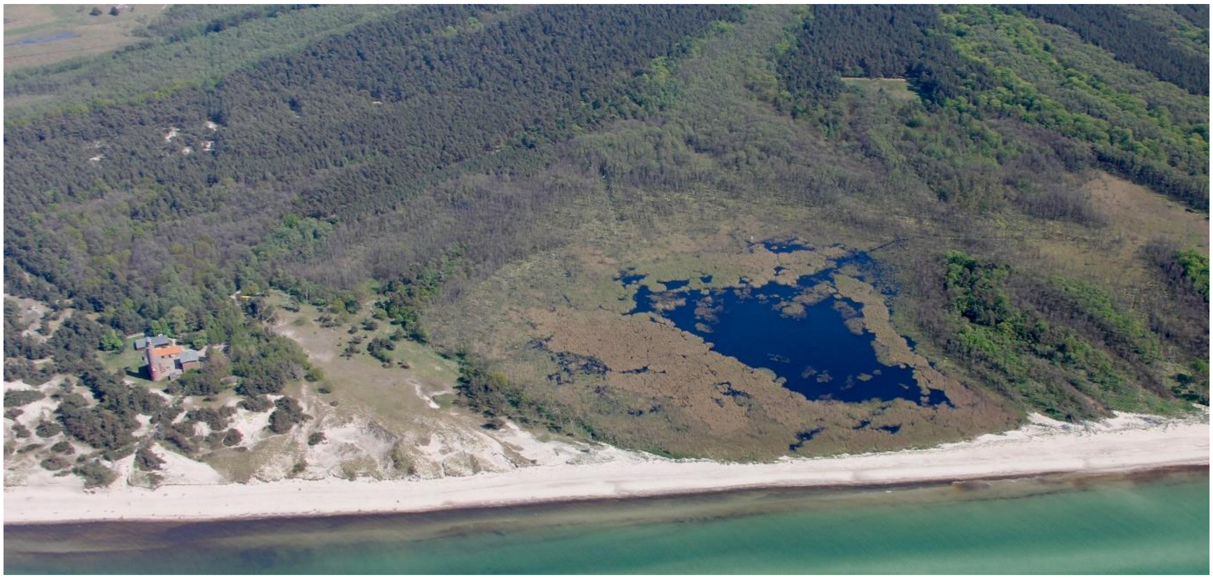

Fig. S9: Aerial photograph of the western location indicated in Fig. 5 from Mai 2013 indicating several dead trees (grey canopies) that died after 2011. Image courtesy of Lutz Storm.

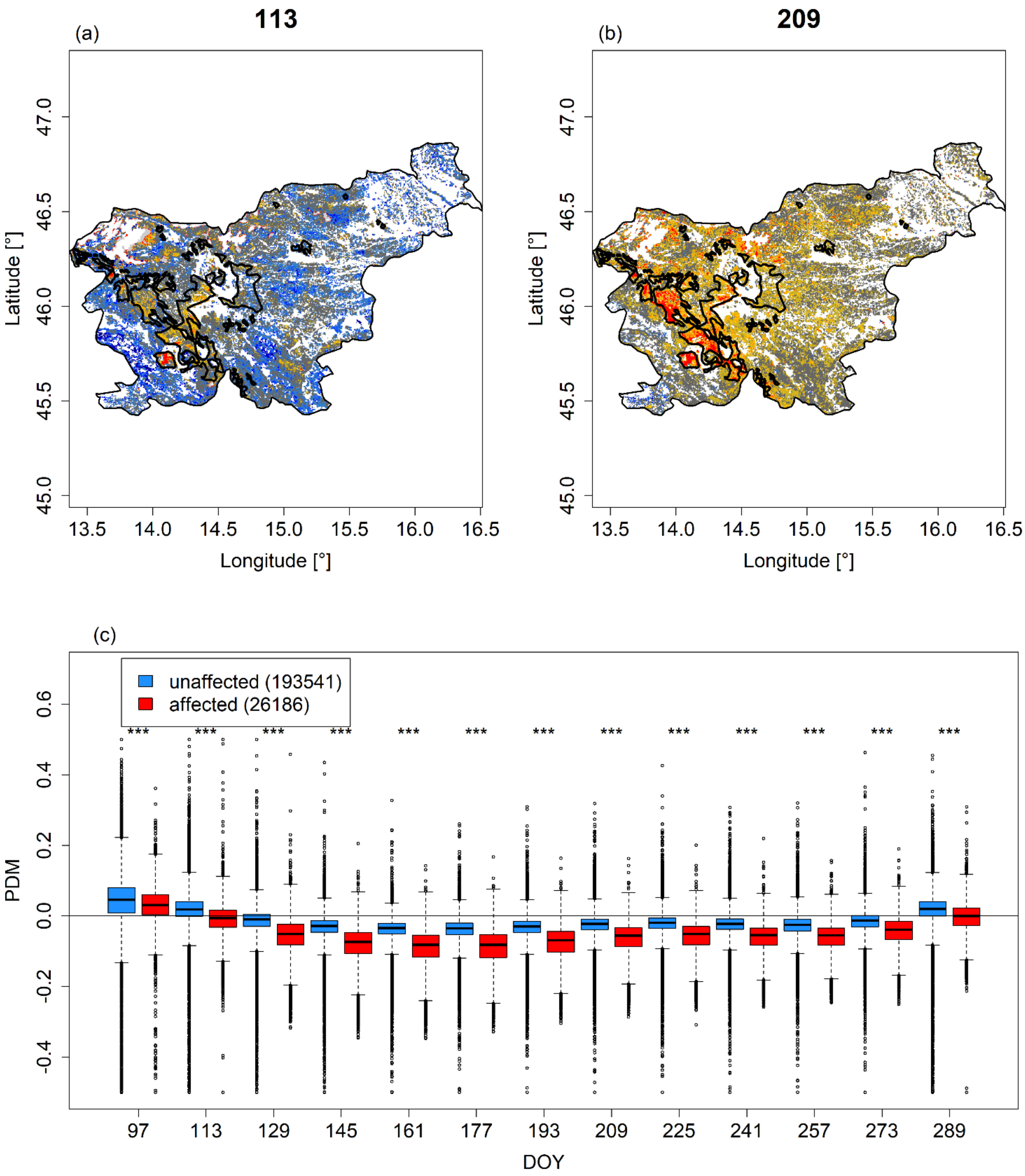

Fig. S10: PDM mapped for Slovenia on DOY 113 (a) and 209 (b). The polygons demarcate areas affected by the ice storm according to Roženbergar et al. (2020). Corresponding values grouped according to affectedness from DOY 97 until DOY 289 are shown in panel (c). The horizontal line in (c) demarcates median *PDM* values of 0. Significance stars indicate significant differences between DOY-specific groups according to Wilcoxon rank-sum test.

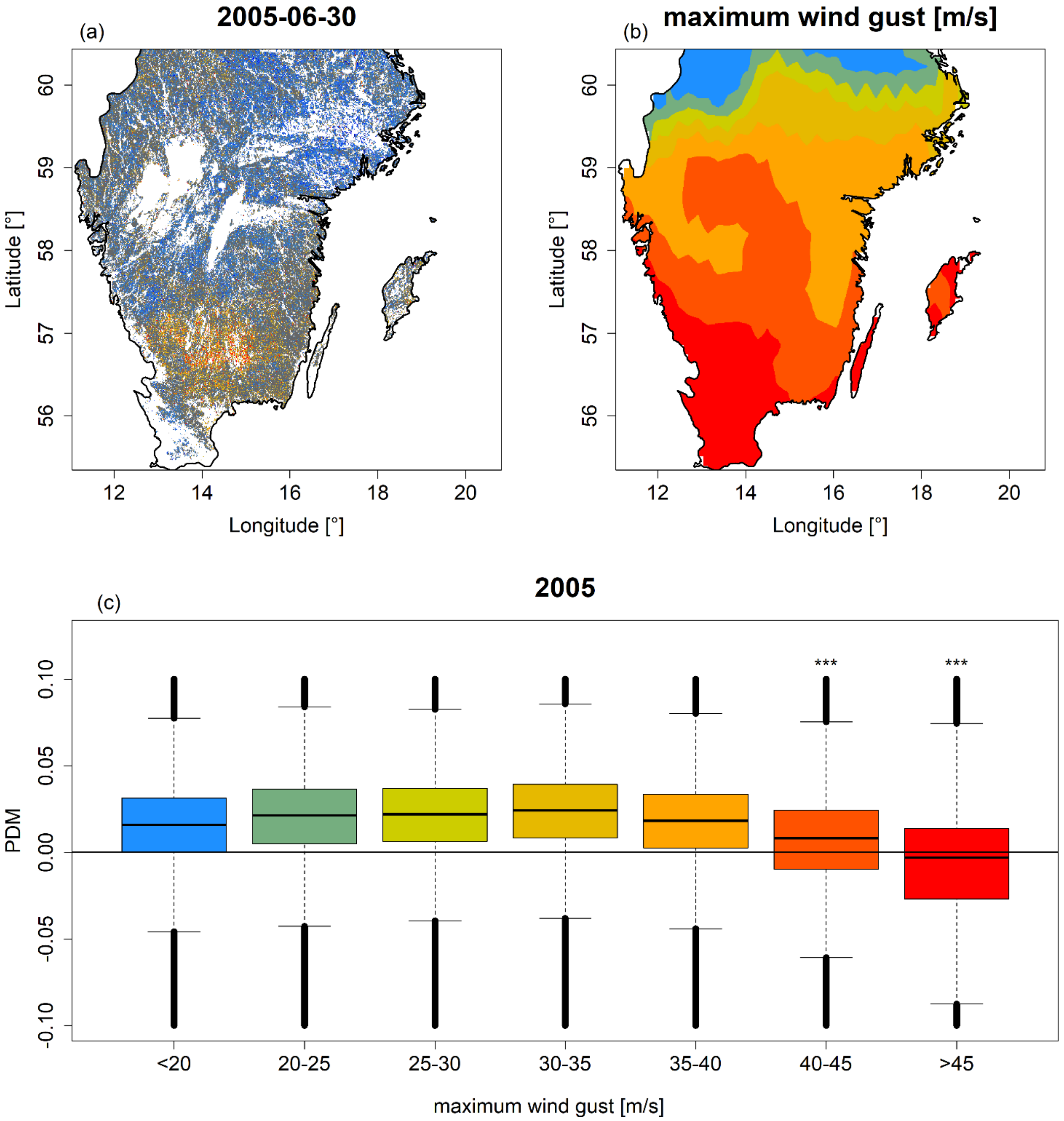

Fig. S11: *PDM* mapped for southern Sweden on 30th of June, 2005 (a) along with maximum wind gust speed during the winter storm 'Gudrun' in January 2005 (b) and boxplots of *PDM* grouped according to different categories of wind gust speed (c). Significance stars in (c) demarcate a significant ($p<0.001$) one-sided Wilcoxon rank-sum test between subtle storm impact (gust speed < 20 m/s) and the corresponding groups. Colors in (b) and (c) refer to the same wind gust speed categories as displayed on the x-axis of (c). The horizontal line in (c) demarcates median *PDM* values of 0. Please note, that the y-axis has been truncated to the range between -0.1 and 0.1, allowing for better recognizing the differences between boxplots.

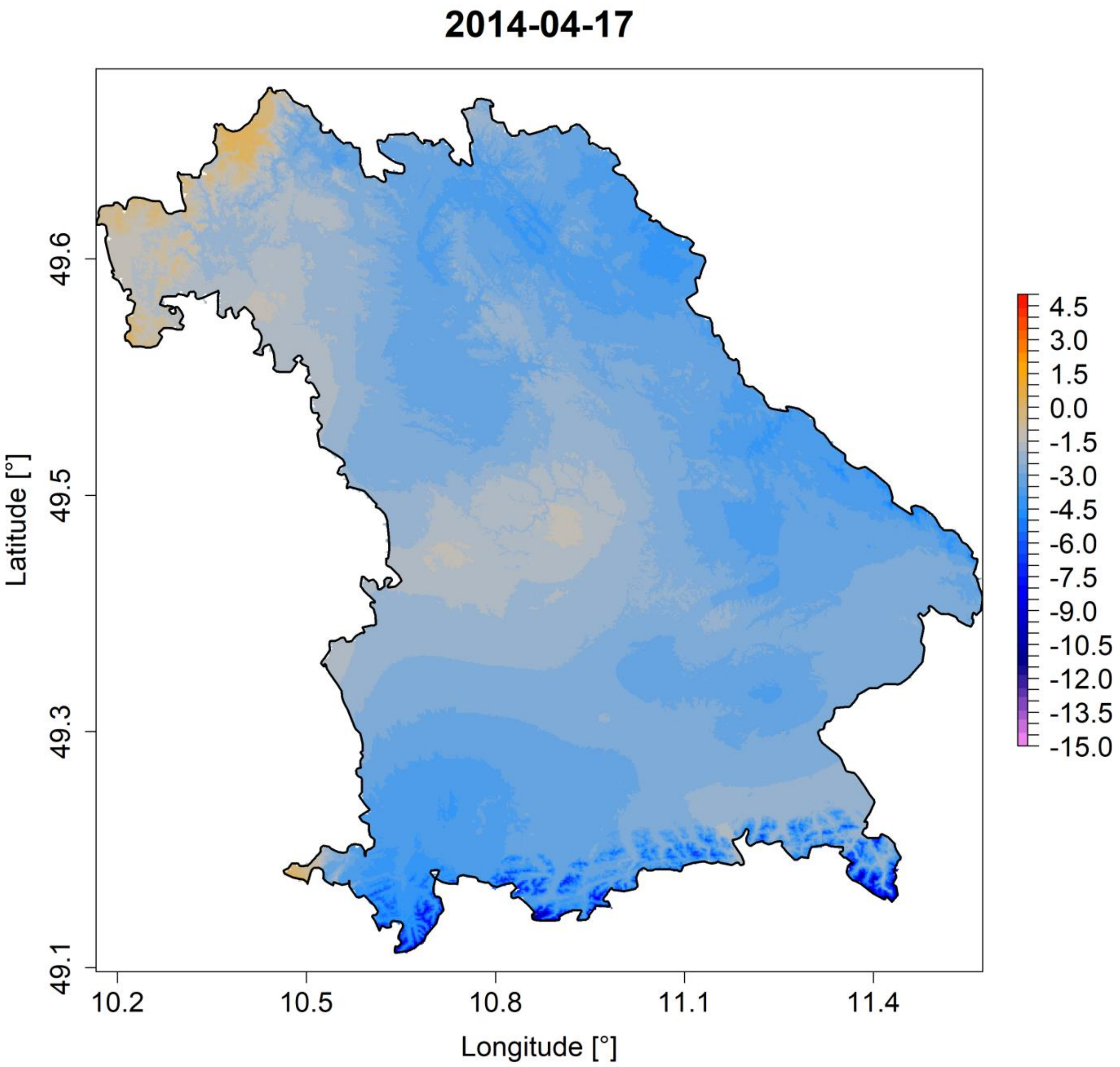

Fig. S12: Thin-plate spline interpolated minimum temperature for April 17th 2014 across Bavaria indicating a potential late-frost event.
