# Supplementary material for: The European Forest Condition Monitor: Using Remotely Sensed Forest Greenness to Identify Hot Spots of Forest Decline
Source: Front Plant Sci. 2021 Dec 1;12:689220. doi: 10.3389/fpls.2021.689220 (PMC8672298; doi:10.3389/fpls.2021.689220)
Supplement: Supplementary file 4 [file Data_Sheet_2.PDF]

###EFCMviewer code

```
ui<-fluidPage(
 titlePanel("European Forest Condition Monitor"),
 sidebarLayout(
  sidebarPanel(width=4,
        dateInput(inputId="date",label="Date 1",
             min="2001-01-01",max="2020-12-31",
             weekstart=1,width='20%',value="2001-07-29"),
        dateInput(inputId="date2",label="Date 2",
             min="2001-01-01",max="2020-12-31",
             weekstart=1,width='20%',value="2020-07-29"),
        selectInput(inputId="product",label="Product",
              choices=c("Quantiles","Proportions"),width='40%',selected="Quantiles"),
        downloadButton("downloadMap1",label="Map 1"),
        downloadButton("downloadMap2",label="Map 2"),
        downloadButton("downloadHist1",label="Histogram 1"),
        downloadButton("downloadHist2",label="Histogram 2"),
        textOutput("reference"),
        textOutput("coordination")
  ),
  mainPanel(width=8,
       plotOutput(outputId="map",height=500,width=800),
       plotOutput(outputId="hist",height=500,width=800)
  )
 )
)
```

```
server<-function(input,output)
{
  FILENAMES<-reactive({
    if(input$product=="Quantiles")
    {OUT<-QUANTNAMES}
    if(input$product=="Proportions")
    {OUT<-PROPNAMES}
    return(OUT)
  })
  FILEHISTNAMES<-reactive({
    if(input$product=="Quantiles")
    {OUT<-QUANTHISTNAMES}
    if(input$product=="Proportions")
    {OUT<-PROPHISTNAMES}
    return(OUT)
  })
  DOYINFO<-reactive({
    if(input$product=="Quantiles")
    {OUT<-substr(QUANTNAMES,6,12)}
    if(input$product=="Proportions")
    {OUT<-substr(PROPNAMES,6,12)}
    return(OUT)
  })
  PRODUCT1<-reactive({
    Selection<-which.min(abs(as.numeric(DOYINFO())-

as.numeric(paste(substr(input$date,1,4),strftime(input$date,format="%j"),sep=""))))
    RAST<-stack(FILENAMES()[Selection])
    return(RAST)
  })
  PRODUCT2<-reactive({
    Selection<-which.min(abs(as.numeric(DOYINFO())-
```

```
as.numeric(paste(substr(input$date2,1,4),strftime(input$date2,format="%j"),sep=""))))
    RAST<-stack(FILENAMES()[Selection])
    return(RAST)
  })
  HIST1<-reactive({
    Selection<-which.min(abs(as.numeric(DOYINFO())-

as.numeric(paste(substr(input$date,1,4),strftime(input$date,format="%j"),sep=""))))
    RAST<-stack(FILEHISTNAMES()[Selection])
    return(RAST)
  })
  HIST2<-reactive({
    Selection<-which.min(abs(as.numeric(DOYINFO())-

as.numeric(paste(substr(input$date2,1,4),strftime(input$date2,format="%j"),sep=""))))
    RAST<-stack(FILEHISTNAMES()[Selection])
    return(RAST)
  })
  output$map<-renderPlot({
    par(mfrow=c(1,2))
    plotRGB(PRODUCT1())
    text(x=1000,y=2300,labels=input$date,cex=2)
    plotRGB(PRODUCT2())
    text(x=1000,y=2300,labels=input$date2,cex=2)
  })
  X.LAB<-reactive({
    if(input$product=="Quantiles"){return(1000)}
    if(input$product=="Proportions"){return(1250)}
  })
  output$hist<-renderPlot({
    par(mfrow=c(1,2))
    plotRGB(HIST1())
```

```
    text(x=X.LAB(),y=1400,labels=input$date,cex=2)
    plotRGB(HIST2())
    text(x=X.LAB(),y=1400,labels=input$date2,cex=2)
  })
  output$reference<-renderText("Requested reference if publishing results based on this download: Buras, A. et al., 2020: Quantifying impacts of the 2018 drought on European ecosystems in comparison to 2003, Biogeosciences, 17, 1655-1672")
  output$coordination<-renderText("Coordination: Allan Buras (allan@buras.eu)")
  output$downloadMap1 <- downloadHandler(
    filename = function() {
      paste(input$product,input$date, "_map.tif", sep = "")
    },
    content = function(file) {
        tiff(width=2000,height=2400,res=300,compression="lzw",file=file)
        plotRGB(PRODUCT1())
        text(x=1000,y=2300,labels=input$date,cex=2)
        dev.off()
    }
  )
  output$downloadMap2 <- downloadHandler(
    filename = function() {
      paste(input$product,input$date2, "_map.tif", sep = "")
    },
    content = function(file) {
      tiff(width=2000,height=2400,res=300,compression="lzw",file=file)
      plotRGB(PRODUCT2())
      text(x=1000,y=2300,labels=input$date2,cex=2)
      dev.off()
    }
  )
  output$downloadHist1 <- downloadHandler(
    filename = function() {
```

```
      paste(input$product,input$date, "_histogram.tif", sep = "")
    },
    content = function(file) {
      tiff(width=2500,height=1500,res=300,compression="lzw",file=file)
      plotRGB(HIST1())
      text(x=X.LAB(),y=1400,labels=input$date,cex=2)
      dev.off()
    }
  )
  output$downloadHist2 <- downloadHandler(
    filename = function() {
      paste(input$product,input$date2, "_histogram.tif", sep = "")
    },
    content = function(file) {
      tiff(width=2500,height=1500,res=300,compression="lzw",file=file)
      plotRGB(HIST2())
      text(x=X.LAB(),y=1400,labels=input$date2,cex=2)
      dev.off()

    }
  )
}


shinyApp(ui=ui,server=server)
```
